# Temperature-Associated Anatomical, Photosynthetic, and Putative GC–MS Metabolomic Profiles in the Arid Species *Nitraria sibirica* Pall. and *Nitraria schoberi* L.

**DOI:** 10.3390/metabo16090646

**Published:** 2026-09-03

**Authors:** Dariga Dairbekova, Nina V. Terletskaya, Aigerim Mamirova, Nazym K. Korbozova, Elvira A. Shadenova, Aizhan S. Mussayeva

**Affiliations:** 1 Faculty of Biology and Biotechnology, Farabi University, Al-Farabi 71/19, Almaty 050040, Kazakhstan; ddayrbekova@list.ru (D.D.); naz-ik@mail.ru (N.K.K.); 2 Institute of Genetic and Physiology, Al-Farabi 93, Almaty 050040, Kazakhstan; shadel08@mail.ru (E.A.S.); aimus_@mail.ru (A.S.M.)

**Keywords:** *Nitraria*, microclones, temperature exposure, anatomy, photosynthetic activity, GC–MS profiling

## Abstract

**Backgrounds**: The present study investigated temperature-associated responses of microclones of two halophytic shrubs, *Nitraria sibirica* Pall. and *Nitraria schoberi* L., exposed to low positive (+3 °C) and elevated (+30 °C) temperatures for 72 h. **Methods**: Temperature-associated responses were investigated by evaluation of leaf anatomy, chlorophyll fluorescence, and GC–MS-based metabolite profiling. **Results**: The results revealed shared and species-specific responses, including changes in leaf anatomical dimensions, maintenance or adjustment of PSII-related fluorescence parameters, and treatment-dependent shifts in the relative composition and diversity of putatively annotated metabolite classes. Metabolomic profiling was performed in both in vitro microclones and soil-acclimatised microclones. In vitro microclones showed more pronounced shifts in the number and relative representation of detected compound classes under temperature exposure, whereas soil-acclimatised microclones demonstrated comparatively greater compositional stability. Comparative analysis of the datasets revealed temperature-associated patterns across anatomical, photosynthetic, and relative metabolomic responses; however, these patterns were interpreted descriptively rather than as direct causal mechanisms. Some detected compounds or compound classes have been associated with biological relevance in previous studies; however, their identity, abundance, and activity require targeted validation. **Conclusions**: Overall, the findings provide a descriptive experimental dataset on temperature-associated anatomical, chlorophyll fluorescence, and relative GC–MS profile changes in *Nitraria* microclones and identify candidate metabolite classes for future targeted validation.

## 1. Introduction

Nowadays, ongoing global climate change processes are expected to lead to the degradation and desertification of more than 90% of the Earth’s land surface by 2050 [1]. Under these conditions, a fundamental question arises: how do plants respond to such challenges when they are unable to change their habitats? Which physiological and biochemical changes occur, what factors contribute to survival, and which plant adaptation strategies can potentially be exploited by humans? Both cold and heat stress disrupt fundamental cellular processes, including membrane stability, photosynthetic electron transport, carbon allocation, and metabolic homeostasis [2,3,4]. In natural environments, plants are exposed to a broad spectrum of stressors of varying intensity, ranging from beneficial eustress to severe distress that may lead to wilting and, ultimately, plant death. In contrast, laboratory-based studies often focus on extreme stress conditions; however, it is moderate stress (eustress) that plays a crucial role in promoting adaptive responses and the development of traits of practical value [5,6].

To survive and adapt, plants restructure their root systems and leaves, remodel membrane architecture, regulate water balance, and activate numerous physiological adaptation pathways [7,8]. However, above all, plant adaptation to changing environmental conditions and protection against adverse impacts are closely linked to the efficiency of solar energy conversion in photosynthetic organs. According to published data, under a wide range of climatic and environmental conditions, plant photosynthetic activity tends to reach maximal possible values [9,10]. Consequently, leaf photosynthetic activity represents a key criterion for assessing plant responses to environmental stress, as it directly determines growth and productivity [11,12].

Light acts as a major regulatory factor triggering the biosynthesis and accumulation of a broad spectrum of biologically active compounds (BACs) in plants [12,13,14]. Evidence from the literature, together with results from our own studies, indicates that negative climate change impacts strongly affect numerous metabolic processes in plants [15,16,17,18]. Plant stress responses are coordinated through a series of signalling processes that activate primary metabolism and supply secondary metabolism with biosynthetic precursors. Secondary metabolism, in turn, is largely responsible for a range of physiological responses, including the regulation of ion and water balance and the limitation of oxidative damage, thereby directly or indirectly contributing to plant tolerance of adverse environmental factors. Accordingly, the production of secondary metabolites is considered an adaptive capacity underlying phenotypic plant responses to stress [19,20].

In recent years, plant secondary metabolites have attracted markedly increased interest because of their significance for plant survival and their potential relevance for medical, food, and cosmetic applications [21,22,23,24,25]. However, when such activities are discussed in metabolite-profiling studies, it is important to distinguish literature-reported biological relevance from experimentally confirmed activity. Plants respond to stress as integrated systems in which anatomical plasticity, photoprotective regulation, and secondary metabolism may co-occur [18,26,27]. Disentangling these relationships remains challenging, particularly in long-lived woody species, which are under-represented in experimental studies of stress physiology compared with herbaceous angiosperms. In this context, increased attention should be directed towards plant species that are inherently adapted to extreme climatic conditions, including succulents, xerophytes, and halophytes.

Members of the family Nitrariaceae represent useful models for studying stress-associated structural, physiological, and metabolomic responses in halophytic shrubs. The genus *Nitraria* comprises approximately 10 species distributed across steppe, semi-desert, and desert regions of Asia, North Africa, Southeastern Europe (Romania), and Australia [28]. In the flora of Kazakhstan, these halophytes are represented by the polymorphic species *Nitraria sibirica* Pall. (*N. sibirica*) and *Nitraria schoberi* L. (*N. schoberi*) [29]. These species are widely distributed in arid and semi-arid regions, intermontane basins, saline deserts, solonetz and solonchak soils, steppes, and salt marshes and have historically been used as ornamental, land reclamation, dye, food, and forage plants. Previous pharmacognostic, phytochemical, and histochemical studies have reported that *N. sibirica* and *N. schoberi* contain groups of biologically relevant compounds, including phenolic compounds, flavonoids, alkaloids, polysaccharides, and sesquiterpene lactones [30,31,32,33,34,35,36]. A relationship between the composition of phenolic compounds in *N. sibirica* leaves and environmental conditions has also been reported, which may be relevant for related species [37]. However, the species-specific relationships among temperature exposure, anatomical traits, photosynthetic responses, and relative metabolomic profiles in *Nitraria* microclones remain insufficiently characterised.

One possible approach to addressing these knowledge gaps involves the use of plant cell, tissue, and organ cultures as controlled model systems for studying stress-associated responses [38,39,40]. Microclones of *N. sibirica* and *N. schoberi* have been proposed as experimental alternatives to plants growing under marginal environmental conditions and experiencing continuous abiotic stress, particularly temperature fluctuations and water deficiency. In vitro technologies allow year-round cultivation and a high level of process standardisation, which is useful for comparing relative anatomical, physiological, and metabolomic responses under defined conditions [39,41,42].

Comprehensive studies on the morphophysiological and metabolic responses of microclones to elevated and low positive temperatures remain limited. Therefore, in the present study, microclones of *N. sibirica* and *N. schoberi*, cultivated both in vitro and under soil-acclimatised conditions, were used as model systems to characterise temperature-associated anatomical, photosynthetic, and relative metabolomic responses. As the results of our previous studies, this integrated framework allows comparison of treatment-associated response patterns across complementary datasets [40]. We hypothesise that the eustress conditions established for this study will allow us to compare the species-specific stress responses of *N. sibirica* and *N. schoberi* microclones and to determine whether controlled eustress can induce beneficial modifications in their anatomical and chemical traits.

Within this context, eustress is considered a short-term, non-lethal stimulus that may modulate relative metabolomic profiles; however, the identity, abundance, and biological activity of detected metabolites require further targeted validation.

## 2. Materials and Methods

### 2.1. Plant Material and Cultivation Conditions

Microclones of *Nitraria sibirica* Pall. (*N. sibirica*) and *Nitraria schoberi* L. (*N. schoberi*) were used as the experimental material. Plant material for the establishment of *N. schoberi* microclones was obtained from the Mangyshlak Experimental Botanical Garden (Aktau, Kazakhstan). *N. sibirica* plants were collected from Buiratau National Park (Akmola Region, Kazakhstan). Microclones were obtained through the activation of pre-existing meristems. Apical dominance was removed by excising the apical stem meristem, and the resulting shoots were micro-dissected under sterile conditions. Explants were initially cultured on hormone-free nutrient media during the establishment phase. The media were based on a modified Murashige and Skoog formulation [43]. After 4 weeks of cultivation, shoots that had reached a length of 1.5–2.5 cm and developed a well-formed leaf apparatus were separated from the explants and transferred to Woody Plant Medium [44]. Cultivation continued until the shoots reached 5–7 cm in length, after which a subset was subjected to temperature treatments, while the remainder was transferred to soil-filled vessels.

The basal media were supplemented with meso-inositol (180 mg L^−1^), sucrose (30 g L^−1^), thiamine, pyridoxine, and nicotinic acid (1.5 mg L^−1^ each), and agar (8 g L^−1^). The pH was adjusted to 5.6–6.0 prior to autoclaving. Depending on the cultivation stage, the media were further supplemented with specific plant growth regulators. For shoot multiplication, cytokinins were added, including 6-benzylaminopurine (BAP; 0.5 mg L^−1^), kinetin (2 mg L^−1^), zeatin (8 mg L^−1^), and thidiazuron (0.5 mg L^−1^). Root induction was performed on media supplemented with indole-3-butyric acid (IBA) at a concentration of 0.1 mg L^−1^. Cultures were maintained in test tubes under controlled environmental conditions at 24–26 °C, with a light intensity of approximately 10,000 lux and an 18 h photoperiod. Explants were subcultured onto fresh nutrient medium every 3–5 days. Prior to each subculture, the cut surfaces of the primary explants were refreshed.

Regenerated plantlets with root lengths of 5–7 cm were carefully removed from culture vessels using sterile forceps. The roots were rinsed in a dilute potassium permanganate (KMnO_4_) solution to remove residual agar and reduce the risk of microbial contamination. Following washing, the plantlets were transplanted into soil substrates designed to simulate natural growth conditions. To maintain high humidity during the initial acclimatisation phase, the plants were covered with polyethylene film. The covering was gradually removed over a period of 1–2 weeks. By the end of this stage, the microclones had reached a height of approximately 10–15 cm.

### 2.2. Experimental Design and Sampling Strategy

The experiment was designed as a comparative factorial study including two *Nitraria* species (*N. sibirica* and *N. schoberi*), two cultivation systems where applicable (in vitro microclones and soil-acclimatised microclones), and three temperature conditions: control, elevated temperature (+30 °C), and low positive temperature (+3 °C). Temperature treatments were applied for 72 h under identical light conditions. Each treatment represented three biological replicates, and each biological replicate consisted of three independent plants. Two types of microclones were subjected to temperature stress: (i) in vitro microclones measuring 5–7 cm in length with a well-developed leaf apparatus; and (ii) microclones acclimatised to soil conditions, approximately 10–15 cm in height.

To investigate plant responses to temperature stress, the microclones were transferred to a climate chamber with controlled environmental conditions. The plants were exposed to either elevated temperature (+30 °C) or low temperature (+3 °C) for 72 h. The selected temperature regime was intended to induce short-term, non-lethal eustress rather than severe irreversible damage. The low-temperature treatment of +3 °C was chosen as a low positive chilling condition that can disturb membrane stability, photosynthetic activity, and metabolic regulation, while avoiding freezing injury and intracellular ice formation [2,3,4]. The +30 °C treatment was selected as a moderate heat-stress condition relevant to the warm-season thermal range experienced by *Nitraria* species in arid and semi-arid habitats [28,29]. This temperature is sufficiently elevated to induce measurable physiological and metabolic responses but does not represent acute heat shock. Temperatures above +30 °C were not used because they could shift the response from adaptive eustress to distress and, under in vitro conditions, may also affect the stability of the agar-based culture medium. The 72 h exposure period was selected as an acute induction window long enough to elicit measurable photosynthetic, anatomical-dimensional, and metabolomic responses while maintaining plant viability and allowing comparison between species and cultivation systems [40]. During the treatment period, light intensity was maintained at approximately 10,000 lux under an 18 h photoperiod. Each experimental treatment was conducted in triplicate, with each biological replicate comprising three independent plants.

Sampling was performed immediately after the 72 h exposure period. Fully developed leaves of comparable developmental stage and position were selected for anatomical measurements, chlorophyll fluorescence analysis, and GC–MS profiling. Anatomical and chlorophyll fluorescence measurements were performed on in vitro-grown microclones, whereas GC–MS profiling was performed on both in vitro-grown and soil-acclimatised microclones. For GC–MS analysis, leaf material from each biological replicate was processed independently. Where plant material from several plants was combined to obtain the required extraction mass, this pooled material represented one biological replicate.

**Figure 1:**
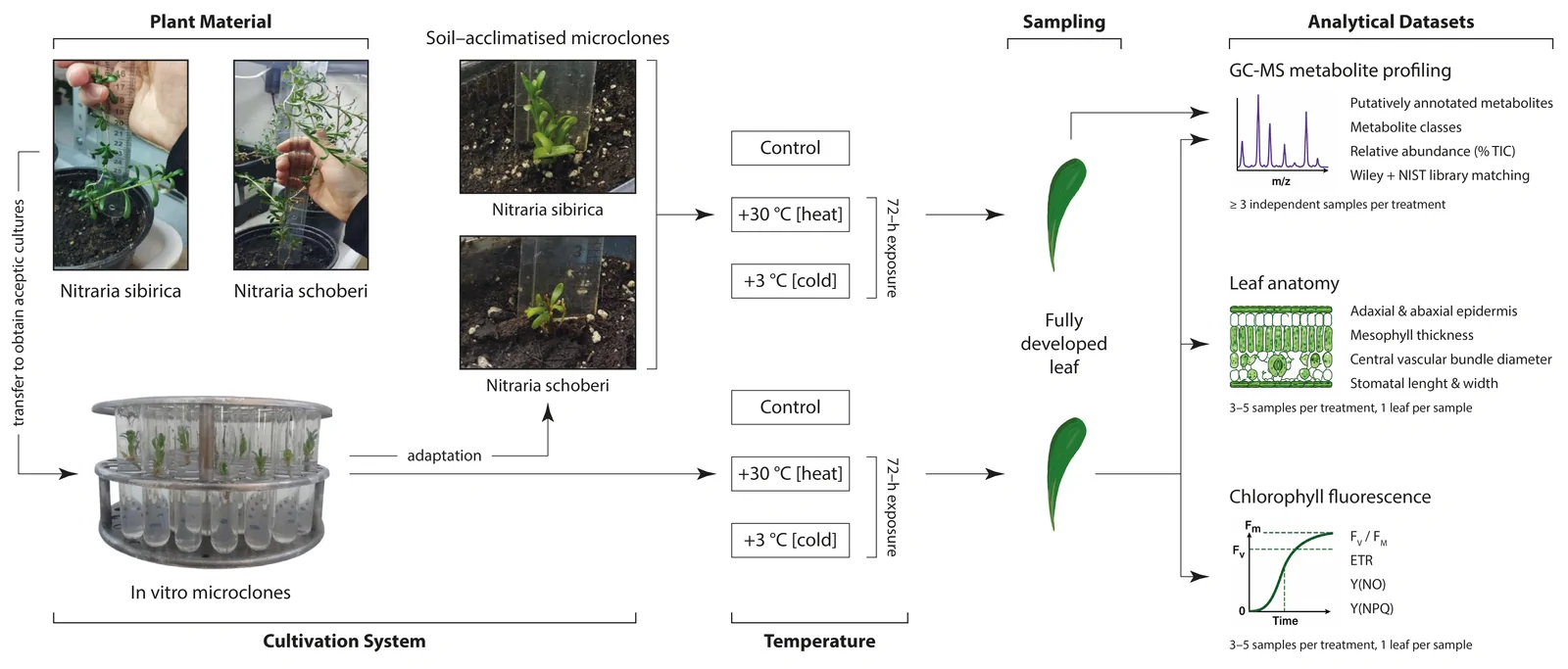
Experimental design, replication structure, and analytical workflow used to evaluate temperature-associated responses in *N. sibirica* and *N. schoberi* microclones.

Biological replicates were treated as independent experimental units. Technical replication was limited to repeated measurements within each biological replicate for anatomical and fluorescence parameters. GC–MS results were interpreted at the level of biological replicate-derived extracts and are reported as relative peak-area-based profiles rather than absolute metabolite concentrations. No causal relationship among anatomical, photosynthetic, and metabolomic parameters was assumed unless directly supported by the experimental design (Figure 1).

### 2.3. Phenotypic Evaluations

#### 2.3.1. Anatomical Structure Assessment

For anatomical analysis, fully developed leaf blades from in vitro-grown *Nitraria* microclones were selected. Leaf samples were fixed in 70% ethanol, and Strasburger–Flemming’s mixture was used as the preservative fluid. The mixture consisted of 96% ethanol, glycerol, and water in a 1:1:1 ratio [45]. Anatomical sections were prepared using an MZP-01 microtome (Technom, Ekaterinburg, Russia), equipped with a freezing unit OL-ZSO 30 (Inmedprom, Yaroslavl, Russia). Microscopic images of the anatomical sections were obtained using a Micro Opix MX 700 (T) microscope (West Medica, Wiener Neudorf, Austria) equipped with a CAM V1200C HD camera (West Medica, Wiener Neudorf, Austria). All anatomical observations were performed using a 40× objective. The analysis included 3–5 replicates, with three plants per replicate.

#### 2.3.2. Photosynthetic Activity Determination

For the assessment of photosynthetic activity, fully developed leaf blades from in vitro-grown *Nitraria* microclones were selected. Photosynthetic activity was assessed by chlorophyll fluorescence measurements. Rapid light curves (RLCs) were recorded using a Junior-PAM fluorometer (Heinz Walz GmbH, Effeltrich, Germany) with actinic illumination at 450 nm. During each measurement, eight saturation light pulses of 10,000 μmol m^−2^ s^−1^ were applied at 20 s intervals, while actinic light intensity was increased stepwise from 0 to 625 μmol m^−2^ s^−1^ [46]. Data used for comparison were obtained from the final saturation pulse of the light curve. At this stage, all analysed parameters reached a plateau, providing stable indicator values. The following parameters were calculated using Win-Control-3.29 software (Walz, Effeltrich, Germany): *F_V_/F_M_*, the maximum quantum yield of PSII photochemistry; *ETR*, the relative electron transport rate of PSII; *Y*(*NO*), the quantum yield of non-photochemical energy dissipation in PSII associated with downregulation of the light-harvesting function; and *Y*(*NPQ*), the quantum yield of non-photochemical energy dissipation in PSII due to regulated energy dissipation. All measurements were performed on the middle third of fully developed leaves. Photosynthetic measurements were conducted with 3–5 replicates, with one leaf analysed per replicate.

#### 2.3.3. Phytochemical Analysis by Gas Chromatography–Mass Spectrometry

For comparative phytochemical analysis, fully developed leaf blades from in vitro-grown *Nitraria* microclones and from microclones acclimatised to soil conditions were selected. For each experimental variant, 10 g of plant material were extracted with 50 mL of 96% ethanol for 30 days. At least three independent replicates were prepared for each variant. GC–MS analysis of *N. sibirica* and *N. schoberi* extracts was performed using an Agilent 7890A/5975C system (Santa Clara, CA, USA). A sample volume of 0.7 μL was injected at an injection temperature of 280 °C. Analyses were performed in splitless mode. Chromatographic separation was carried out on a DB-17MS capillary column (30 m length, 0.25 mm inner diameter, 0.25 μm film thickness). Helium was used as the carrier gas at a constant flow rate of 1 mL min^−1^. The oven temperature programme started at 40 °C with no initial hold. The temperature was increased to 280 °C at a rate of 5 °C min^−1^, followed by a final hold at 280 °C for 10 min. The total run time was 58 min. Mass spectrometric detection was performed in SCAN mode over an *m*/*z* range of 34–750. The GC–MS system was controlled using Agilent MSD ChemStation software (v. 1701EA, Santa Clara, CA, USA). The software was used for data acquisition and processing. Data processing included determination of retention times, analysis of peak areas, and processing of mass spectra. Compound identification was performed using the Wiley 7th edition and NIST’02 mass spectral libraries, containing more than 550,000 spectra.

Relative abundance was estimated using peak-area normalisation and expressed as a percentage of the total ion current/total ion chromatogram area. As these values represent compositional data, they were interpreted as relative peak-area-based profiles rather than absolute concentrations [47,48,49]. Compound annotation was based on comparison of mass spectra with the Wiley 7th edition and NIST’02 spectral libraries. Because authentic standards, targeted quantification, retention-index confirmation, and orthogonal analytical confirmation were not used, detected compounds are reported as putatively annotated metabolites or compound classes. The level of interpretation therefore follows a conservative spectral-library-based annotation framework consistent with metabolomics reporting recommendations and confidence-based reporting principles [50,51]. Metabolite identities and biological activities require future validation using authentic standards, targeted GC–MS/LC–MS methods, MS/MS confirmation where applicable, retention-index verification, absolute quantification, and functional bioactivity assays.

#### 2.3.4. Analytical Traceability and Annotation Confidence

To support independent evaluation of the GC–MS observations, representative total ion chromatograms (TICs) for each species, cultivation system, and temperature treatment are provided in the Supplementary Material (Figures S1 and S2). A peak-level annotation and traceability table accompanies the chromatograms and includes sample/variant identifier, species, cultivation system, treatment, retention time, putative compound or compound class, relative peak area (% TIC), and library-matching information where available (Tables S1 and S2). Because the supplied processed peak tables do not contain replicate-level raw peak areas, this limitation is explicitly stated in Tables S1 and S2. If library similarity scores or retention indices are unavailable for some peaks, this is also stated explicitly in the supplementary table. Because the present workflow did not include authentic standards, targeted quantification, retention-index confirmation, or orthogonal analytical confirmation, metabolomic results are interpreted at the level of relative chromatographic profiles and putatively annotated compound classes rather than confirmed compounds or absolute concentrations.

An evidence-level summary linking each dataset to its supported and unsupported interpretations is also provided in Supplementary Table S3. In the present manuscript, anatomical, chlorophyll fluorescence, and GC–MS datasets are used to describe co-occurring treatment-associated response patterns. They are not used to infer direct causal relationships among anatomical modification, photosynthetic regulation, and metabolite profiles.

### 2.4. Data Integration and Statistical Analysis

Anatomical, chlorophyll fluorescence, and GC–MS datasets were integrated at the treatment-group level to identify concordant or divergent response patterns between species, cultivation systems, and temperature treatments. The cross-dataset interpretation was restricted to descriptive co-occurrence of response patterns. Direct causal relationships among anatomical modification, photosynthetic regulation, and metabolite profiles were not inferred from the available design. Statistical analyses were performed using RStudio software (version 2023.06.0, build 421; RStudio PBC, 2023). Analysis of variance (ANOVA; one- or two-way) was used to identify statistically significant differences among experimental variants. When significant effects were detected by ANOVA, Tukey’s honestly significant difference (HSD) test was applied for pairwise comparisons. Experimental variants were classified according to the test results using letter-based groupings in descending order at *p* < 0.05. Hierarchical cluster analysis was performed using the “dendextend” and “pheatmap” libraries. The evidence level and interpretation limits for each dataset are summarised in Table S3.

## 3. Results

A comparative analysis of in vitro-cultured *N. sibirica* and *N. schoberi* microclones was conducted to describe anatomical, chlorophyll fluorescence, and relative metabolomic responses to elevated and low positive temperature exposure.

### 3.1. Stress-Induced Anatomical Alterations in N. sibirica and N. schoberi Microclones

**Figure 2:**
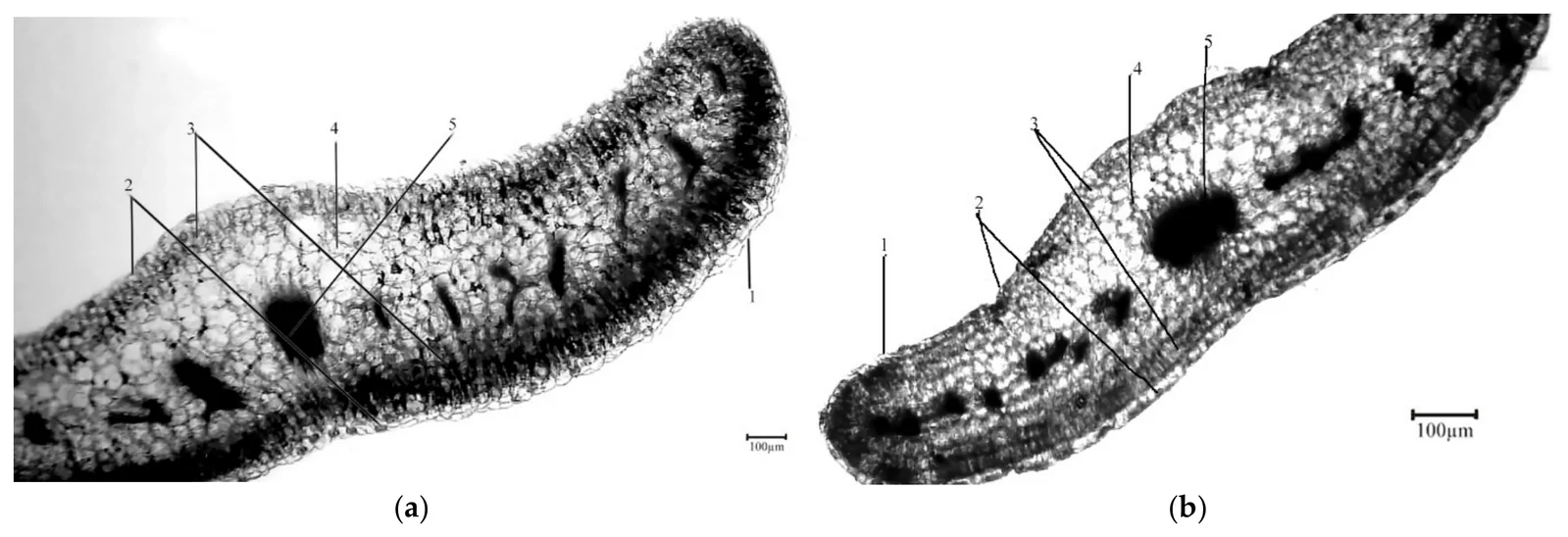
Cross-section of a leaf: (**a**) *N. sibirica*; (**b**) *N. schoberi*. Notes: 1—thin cuticle; 2—epidermis; 3—columnar mesophyll; 4—spongy mesophyll; 5—central vascular bundle.

The leaves of both studied species were flat, isolateral, and amphistomate (Figure 2). In both *Nitraria* species, the adaxial and abaxial epidermis consisted of oblong and rounded cells.

**Figure 3:**
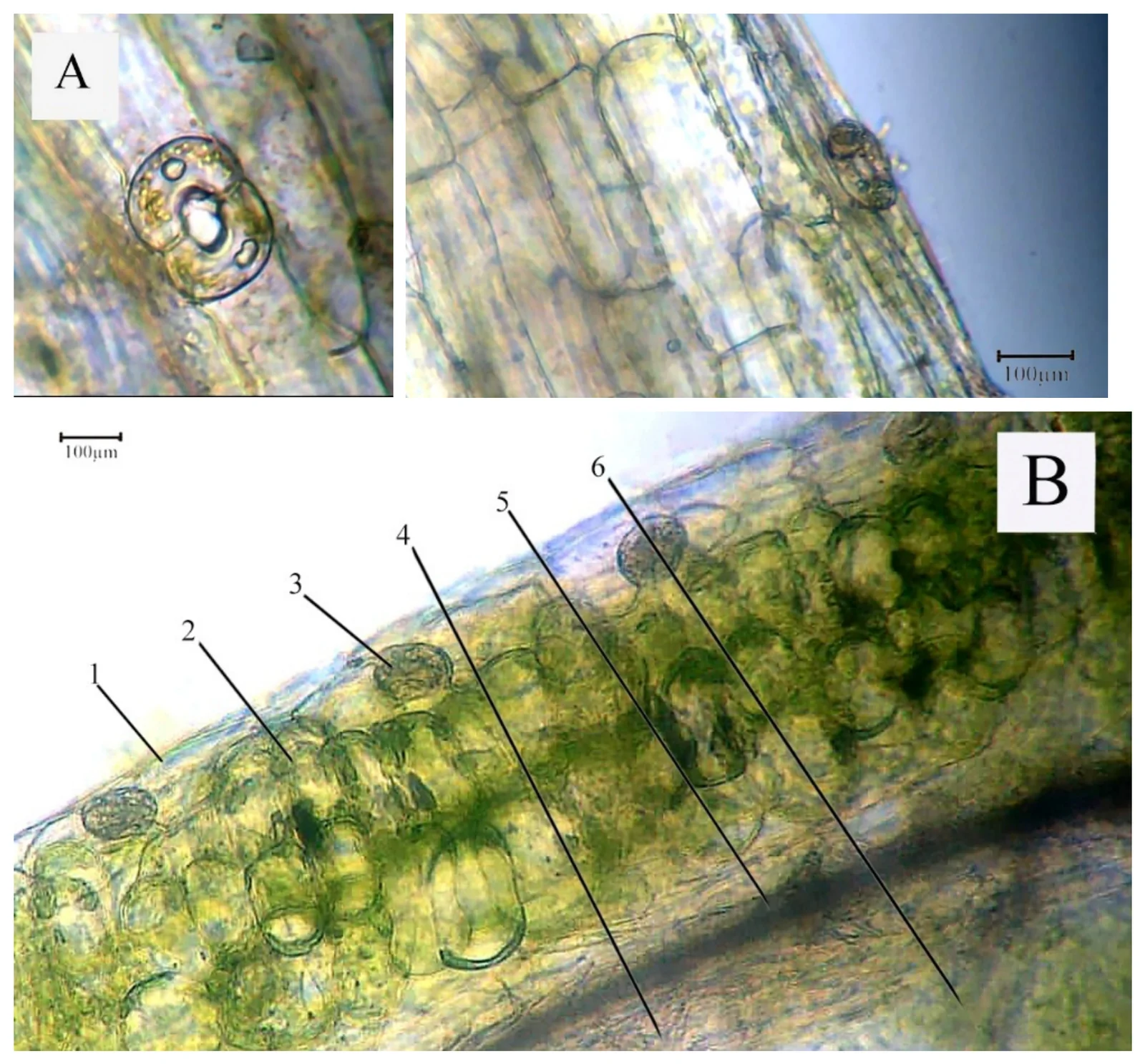

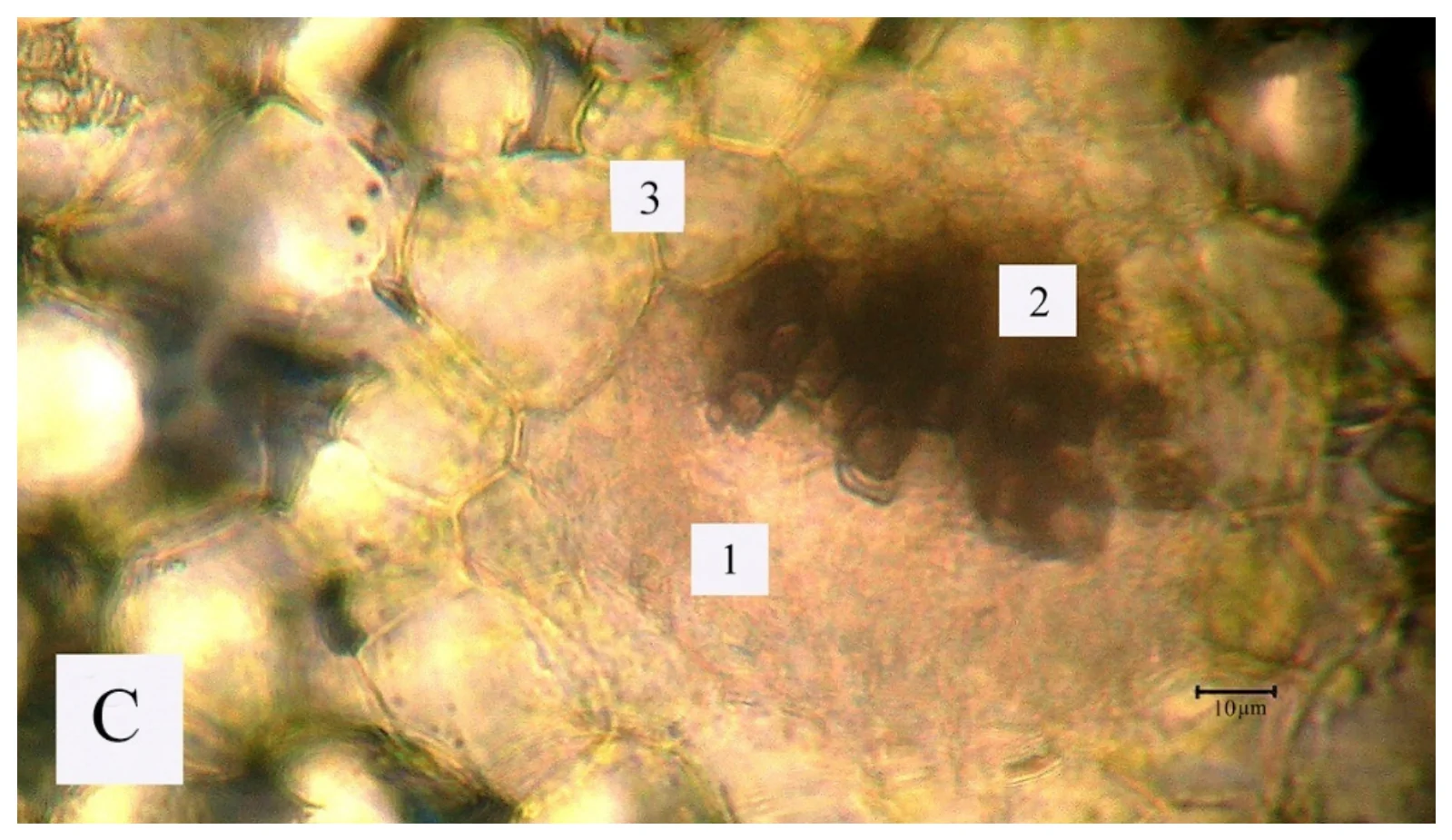
Anatomical structures of the *Nitraria* leaf. (**A**) stoma not immersed in the mesophyll; (**B**) structural elements of the leaf (1—epidermis; 2—palisade mesophyll; 3—glandular idioblasts; 4—sclerenchyma; 5—vessel; 6—spongy mesophyll); (**C**) vascular bundle (1—phloem; 2—xylem; 3—sclerenchyma).

The epidermal surface was covered with sparse, unicellular, simple trichomes. Numerous stomata were present on both sides of the leaf blade and were not immersed in the epidermis. The stomata were oval and of the anomocytic type (Figure 3A). In addition, orderly chloroplast structures are visible along the cell walls of the mesophyll cells (Figure 3A). The mesophyll was differentiated into several irregular rows of palisade tissue (columnar mesophyll) oriented towards both leaf surfaces, with spongy tissue located between them. Cells of the spongy tissue were isodiametric, spherical, elongated, or irregular in shape. The mesophyll was penetrated by small vascular bundles (Figure 3B). Glandular idioblasts were observed, most of which were located adjacent to the epidermal layers (Figure 3B). The central vascular bundle was rounded and consisted of xylem and phloem strands surrounded by a sclerenchyma sheath (Figure 3C).

Exposure of *N. sibirica* and *N. schoberi* microclones to high and low positive temperatures in vitro did not result in visible changes in leaf lamina anatomy; therefore, the presentation of comparative images was considered unnecessary. However, significant adaptive changes in the dimensions of individual anatomical structures were detected (Table 1).

Under standard (control) growth conditions, *N. schoberi* exhibited a slightly thinner adaxial and abaxial epidermis than *N. sibirica*, by 3.69% (*p* < 0.05) and 4.49% (*p* < 0.01), respectively. In contrast, *N. sibirica* was characterised by a larger diameter of the central vascular bundle (by 19.7%, *p* < 0.001) and greater stomatal length and width (by 13.9% (*p* < 0.001) and 7.20% (*p* < 0.001), respectively) compared with *N. schoberi*.

Exposure to high temperatures caused a statistically significant thickening of both the adaxial and abaxial epidermis in *N. sibirica* leaves (by 4.51% and 3.67%, respectively), whereas a reduction in abaxial epidermis thickness was observed in *N. schoberi* leaves (by 6.64%). High-temperature treatment also resulted in a reduction in the diameter of the central vascular bundle and a decrease in stomatal width in both *Nitraria* species, by 8.57–12.8% and 54.7–69.5%, respectively (Table 1). With respect to stomatal length, exposure to high temperature led to a decrease in *N. sibirica* (by 13.0%), whereas an increase was observed in *N. schoberi* (by 8.33%). Mesophyll thickness in both species was not affected by high-temperature exposure.

Exposure to low temperatures resulted in a decrease in mesophyll thickness in *N. sibirica* (by 6.63%) but an increase in this parameter in *N. schoberi* (by 7.42%). In *N. sibirica* microclones exposed to low temperature, all analysed anatomical parameters showed reductions, including adaxial epidermis (by 3.28%), abaxial epidermis (by 1.63%), mesophyll (by 6.63%), diameter of the central vascular bundle (by 7.14%), stomatal length (by 13.0%), and stomatal width (by 59.3%). In contrast, structural responses in *N. schoberi* microclones were more heterogeneous. Low-temperature exposure did not affect adaxial epidermis thickness but resulted in increases in abaxial epidermis thickness (by 7.03%), mesophyll thickness (by 7.42%), central vascular bundle diameter (by 2.56%), and stomatal length (by 8.33%), accompanied by a reduction in stomatal width (by 53.4%).

Based on these observations, the following response patterns were identified: (1) reduction in stomatal width was observed as a shared stress response in both species under both temperature treatments; (2) reductions in stomatal length and central vascular bundle diameter represented a shared response specific to *N. sibirica*; (3) an increase in stomatal length represented a shared response specific to *N. schoberi*; and (4) mesophyll thickness responded specifically to low-temperature exposure in both species, with opposite trends, showing thinning in *N. sibirica* and thickening in *N. schoberi*.

### 3.2. Stress-Induced Alterations in Photosynthetic Activity in N. sibirica and N. schoberi Microclones

**Figure 4:**
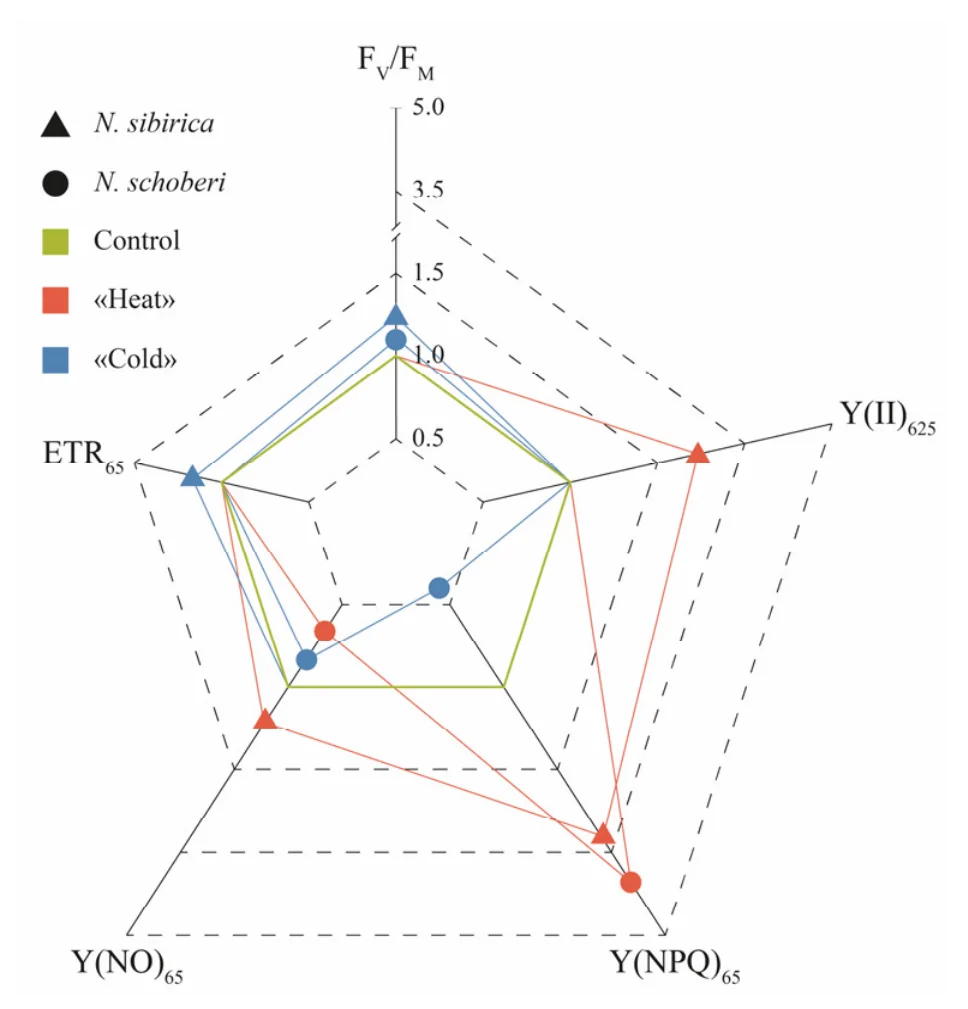
Changes in stress-induced photosynthetic activity in plant leaves.

Comparison of photosynthetic behaviour between the studied species showed that, under standard (control) growth conditions, *N. sibirica* exhibited higher *ETR*_65_ values (by 10.3%, *p* < 0.05), whereas *N. schoberi* showed higher *F_V_/F_M_* and *Y*(*NO*)_65_ values by 6.45% (*p* < 0.05) and 21.9% (*p* < 0.001), respectively (Table S4). Under high-temperature stress, *F_V_/F_M_* and *ETR*_65_ values remained stable in both species (Figure 4). High-temperature exposure was associated with an increase in *Y*(*NPQ*)_65_ values in both species, indicating enhanced non-photochemical energy dissipation. Specifically, *Y*(*NPQ*)_65_ increased by 3.33-fold in *N. sibirica* and by 4.07-fold in *N. schoberi* (Figure 4). In *N. sibirica*, high-temperature treatment was accompanied by concurrent increases in *Y*(*II*)_625_ and *Y*(*NO*)_65_. In contrast, photosynthetic performance in *N. schoberi* remained comparatively stable under high temperature, and a slight decrease in *Y*(*NO*)_65_ was observed.

Under low-temperature stress, the photosynthetic apparatus of *N. sibirica* remained relatively stable, with slight increases in *ETR_65_* and *F_V_/F_M_*. In *N. schoberi*, exposure to low temperature resulted in more pronounced changes in chlorophyll fluorescence parameters, including decreases in *Y*(*NPQ*)_65_ and *Y*(*NO*)_65_, accompanied by an increase in *F_V_/F_M_*. Based on these results, the following response patterns were identified: (1) an increase in *F_V_/F_M_* values under low-temperature conditions in both species; (2) increases in *Y*(*NPQ*)_65_ and *Y*(*II*)_625_ values under high-temperature conditions in both species; (3) a reduction in *Y*(*NO*)_65_ values as a response characteristic of *N. schoberi* under both temperature treatments.

### 3.3. Stress-Induced Alterations in Metabolomic Profiles of N. sibirica and N. schoberi

Comparative analysis of the relative GC–MS profiles showed that both species exhibited a broader diversity of putatively annotated compound classes when grown in soil compared with in vitro cultures (Tables S5–S8). At the same time, the relative distribution of annotated peak areas differed markedly between cultivation systems. In in vitro cultures, the metabolomic profiles of both species comprised four compound classes, three of which were shared (Tables S5 and S6). Specifically, both species showed comparable relative abundance of fatty acids and their esters (FAEs; *N. sibirica*, 68.1%; *N. schoberi*, 80.0%), organoheterocyclic compounds (OHCCs; *N. sibirica*, 9.44%; *N. schoberi*, 6.39%), and terpenes (*N. sibirica*, 14.8%; *N. schoberi*, 12.0%). The fourth compound class differed between species: *N. sibirica* contained carboxylic acids and their derivatives (CADs; 7.66%), whereas *N. schoberi* contained flavonoids (1.59%).

Under soil-grown cultures, the metabolomic profile of *N. sibirica* comprised 12 classes of compounds, whereas that of *N. schoberi* comprised 7 classes of compounds. All seven compound classes detected in *N. schoberi* were also present in *N. sibirica*, including CADs, FAEs, flavonoids, OHCCs, phthalic acid esters (PhAEs), phenolic compounds (PCs), and terpenes. The relative abundance of flavonoids and PhAEs was comparable between the two species (1.93–2.65% and 1.87–2.96%, respectively). In contrast, the relative abundance of CADs, FAEs, and PCs was higher in *N. schoberi* by 9.22-, 1.54-, and 2.16-fold, respectively, compared with *N. sibirica*. Conversely, the metabolomic profile of *N. sibirica* contained higher proportions of OHCCs and terpenes, by 4.86-fold and 1.38-fold, respectively, relative to *N. schoberi* (Tables S7 and S8).

Comparison of metabolomic profiles between growth systems revealed the following patterns. In *N. sibirica*, in vitro and open-ground cultivation resulted in comparable levels of CADs (7.66% and 8.41%, respectively), accompanied by a 4.19-fold reduction in FAEs relative abundance and increases of 1.51- and 2.06-fold in OHCCs and terpenes, respectively. In *N. schoberi*, comparison of in vitro and soil conditions revealed increases of 1.67-fold and 1.35-fold in flavonoids (1.59% and 2.65%, respectively) and terpenes (12.0% and 16.2%, respectively) relative abundance, together with reductions of 1.54-fold and 1.59-fold in FAEs (80.0% and 25.0%, respectively) and OHCCs (6.39% and 4.01%, respectively) relative abundance.

**Figure 5:**
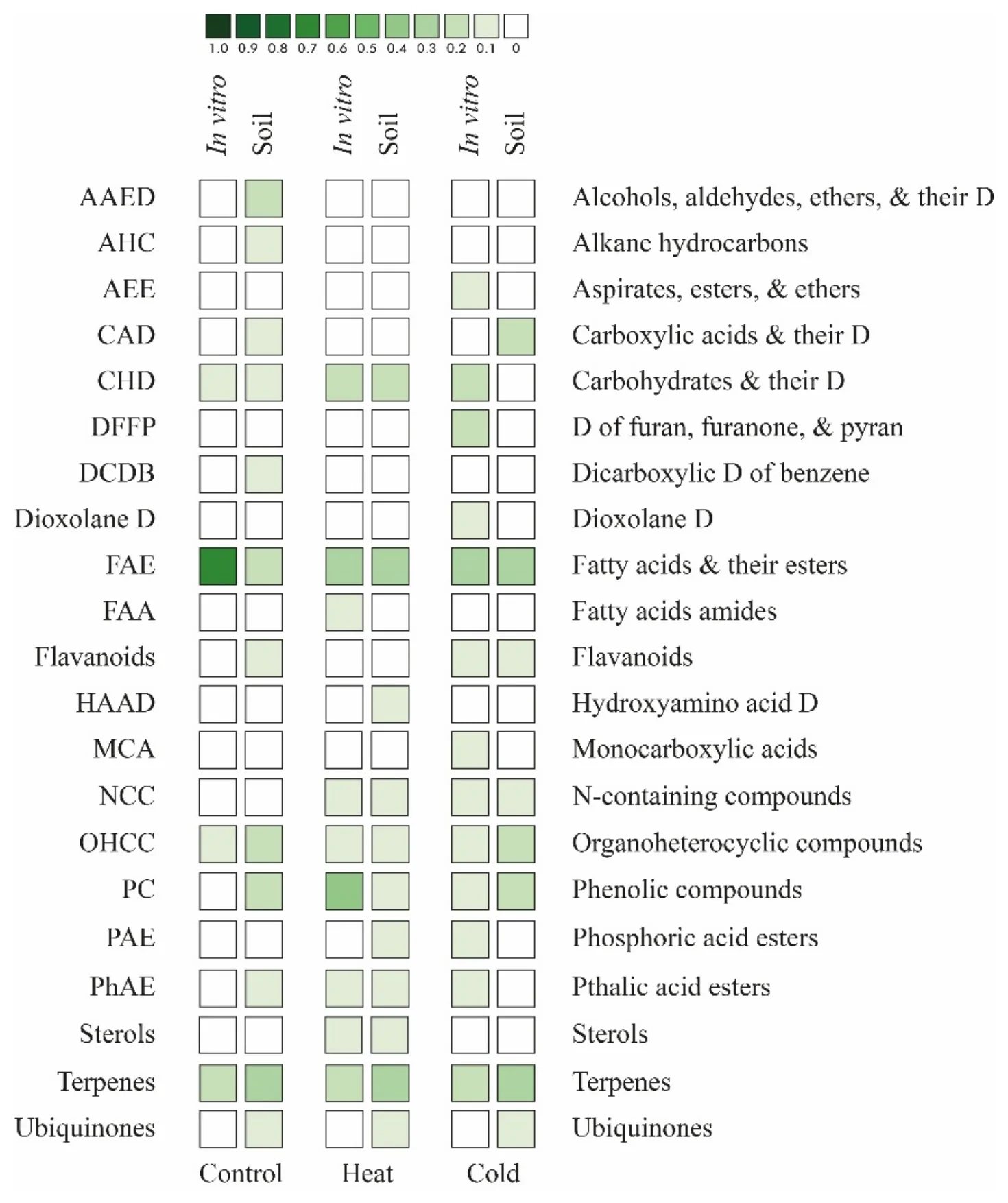
Metabolomic profile of *N. sibirica* exposed to different temperature treatments.

Under high-temperature conditions, the metabolomic profile of *N. sibirica* cultivated in vitro expanded from 4 to 9 compound classes of compounds, whereas in soil-grown plants the profile was reduced from 12 to 11 compound classes (Figure 5). Similarly, exposure to low temperature resulted in an expansion of the in vitro metabolomic profile of *N. sibirica* to 13 classes of compounds, while the diversity of soil-grown plants decreased to 8 classes of compounds. Considering the four compound classes detected in the in vitro metabolomic profile of *N. sibirica* under control conditions, the relative abundance of OHCCs and terpenes was not affected by either high- or low-temperature exposure and fluctuated within the ranges of 8.20–9.50% and 12.9–14.8%, respectively. In contrast, the relative abundance of FAEs decreased markedly under both temperature treatments, from 68.1% to 20.5–24.3%. With respect to CHDs, low-temperature exposure had a more pronounced effect, increasing their relative abundance from 7.66% to 17.7%, whereas high-temperature exposure resulted in an increase to 11.2%.

**Figure 6:**
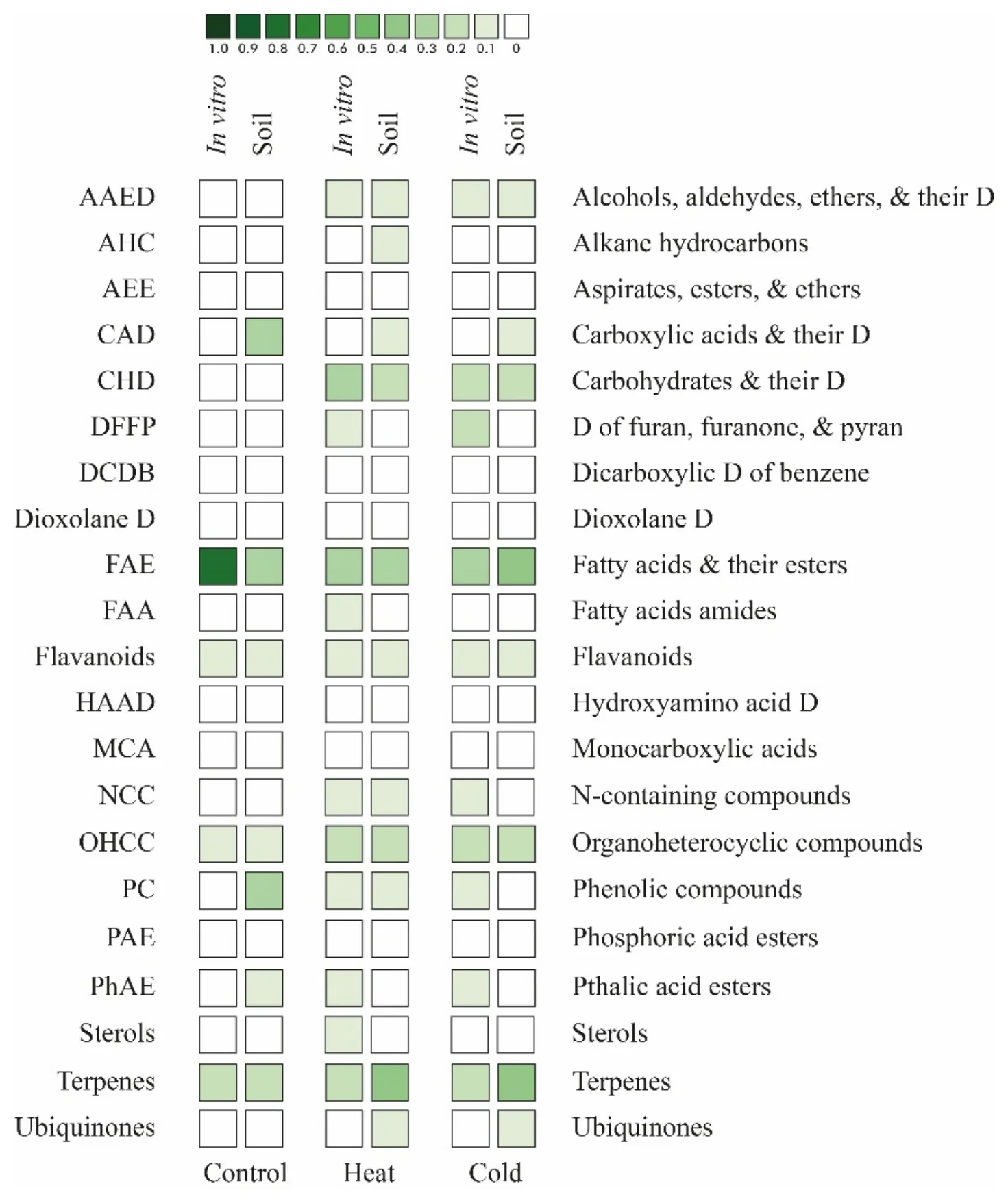
Metabolomic profile of *N. schoberi* exposed to different temperature treatments.

In soil cultures of *N. sibirica*, exposure to high temperature resulted in the absence of detectable CADs and flavonoids from the metabolomic profile, whereas exposure to low temperature led to the absence of CHDs and PhAEs (Figure 6). A common metabolomic response to both temperature treatments was observed in the form of increased relative abundance of FAEs (by 1.52–1.65-fold) and terpenes (by 1.25–1.38-fold), together with a reduction in ubiquinone relative abundance (by 1.47–1.63-fold), with comparable magnitudes under both temperature conditions. A reduction in OHCC relative abundance was also common to both temperature treatments; however, this decrease was more pronounced under high-temperature exposure (by 3.05-fold) than under low-temperature exposure (by 1.82-fold).

Under high-temperature conditions, the metabolomic profile of *N. schoberi* cultivated in vitro expanded from 4 to 12 compound classes, whereas in soil cultures it increased from 7 to 10 compound classes (Figure 6). Similarly, exposure to low temperature resulted in an expansion of the in vitro metabolomic profile of *N. schoberi* to 10 compounds, while the diversity of soil-grown plants increased by one compound class, to 8. Considering the four classes of compound classes detected in the in vitro metabolomic profile of *N. schoberi* under control conditions, terpene relative abundance was not affected by either high- or low-temperature exposure and fluctuated within the range of 11.5–13.3%. In contrast, the relative abundance of FAEs decreased markedly under both temperature treatments, from 80.0% to 20.0–24.2%. Unlike *N. sibirica*, the metabolomic responses of *N. schoberi* to eustress showed similar magnitudes under both temperature treatments, with metabolite relative abundance increasing or decreasing to comparable extents. Accordingly, flavonoid relative abundance decreased by 1.75- and 1.79-fold, while relative abundance of OHCCs increased by 2.34- and 2.43-fold under high- and low-temperature stress, respectively.

In soil-grown *N. schoberi*, exposure to low temperature resulted in the absence of detectable PCs from the metabolomic profile. Consistent with the similar responses of *N. schoberi* to high- and low-temperature stress observed under in vitro conditions, comparable metabolomic response patterns were also detected under soil-grown conditions, indicating the scalability of the response from in vitro to soil cultivation systems. Accordingly, exposure of soil-grown *N. schoberi* to either high or low temperature led to the appearance of alcohols, aldehydes, ethers, and their derivatives (AAEDs; 2.04% and 2.09%, respectively), CHDs (15.0% and 12.2%, respectively), and ubiquinones (1.65% and 1.84%, respectively).

Similarly, the relative abundance of OHCCs and terpenes increased under both temperature treatments, from 4.01% to 13.1–16.3% and from 16.2% to 33.5–35.5%, respectively. These changes corresponded to increases of 3.26–4.06-fold for OHCCs and 2.07–2.19-fold for terpenes under both stress conditions. The relative abundance of CADs was reduced under temperature stress; however, the reduction was more pronounced under high-temperature exposure (24.9-fold) than under low-temperature exposure (10.4-fold). PCs also represented a shared stress response in *N. schoberi*, showing a 17.4-fold reduction under high-temperature stress and complete disappearance under low-temperature stress.

### 3.4. Hierarchical Cluster Analysis of Stress-Induced Metabolomic Responses

**Figure 7:**
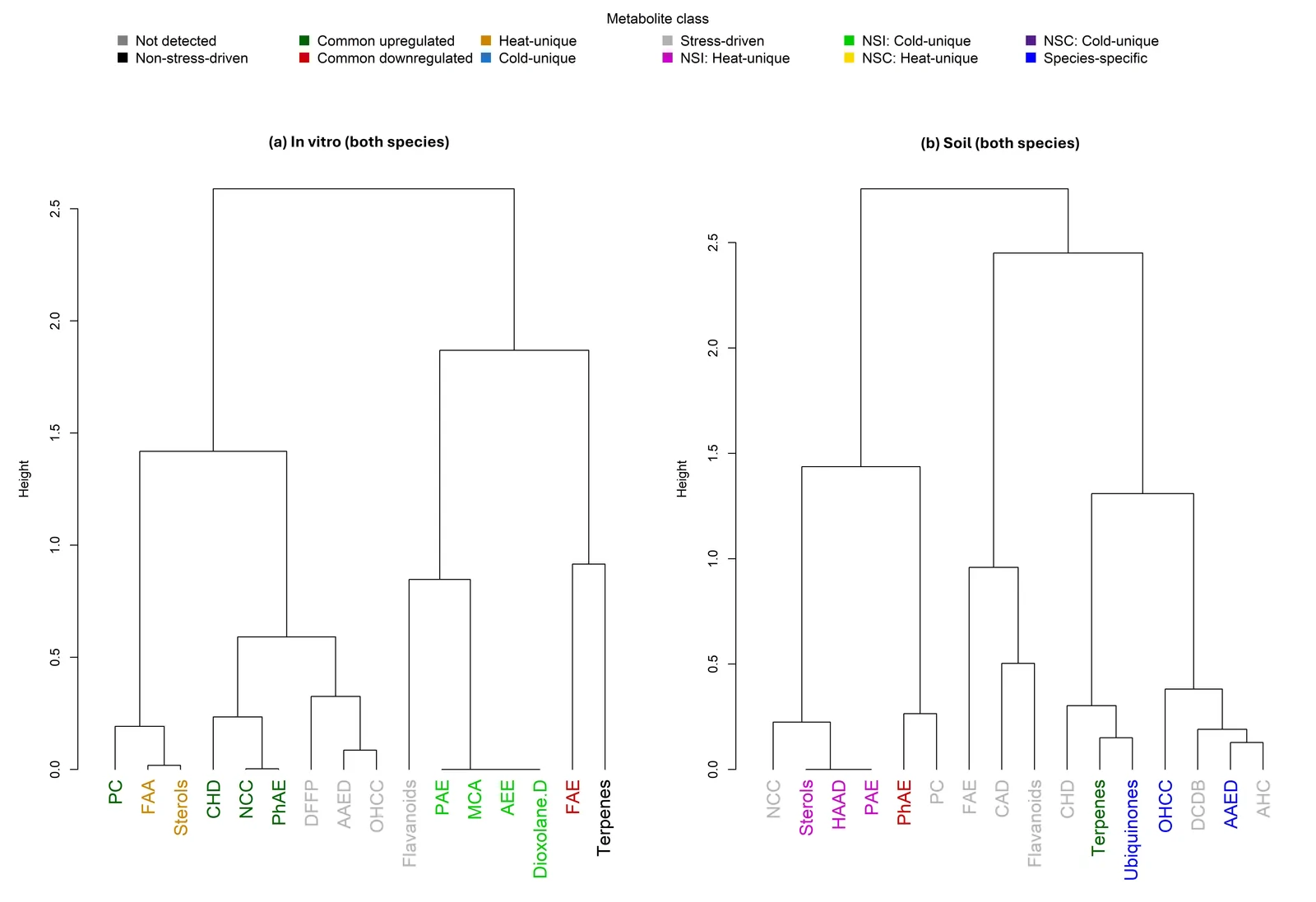
Dendrogram of hierarchical cluster analysis: (**a**) in vitro cultures; (**b**) soil-grown cultures. Notes: NSI—*N. sibirica*; NSC—*N. schoberi*; AAEDs—alcohols, aldehydes, ethers and their derivatives; AEEs—aspirates, esters and ethers; AHCs—alkane hydrocarbons; CADs—carboxylic acids and their derivatives; CHDs—carbohydrates and their derivatives; DCDB—dicarboxylic derivatives of benzene; DFFP—derivatives of furan, furanone and pyran; Dioxlane Ds—dioxolane derivatives; FAEs—fatty acids and their esters; FAAs—fatty acid amides; HAADs—hydroxyamino acids derivatives; MCAs—monocarboxylic acids; NCCs—nitrogen-containing compounds; OHCCs—organoheterocyclic compounds; PAEs—phosphoric acid esters; PhAEs—phthalic acid esters; PCs—phenolic compounds.

Hierarchical cluster analysis was performed based on the following classification criteria (Figure 7): *not detected*—metabolites not detected in either species; *non-stress-driven*—metabolites showing no response in either species under both stress conditions; *common upregulated*—metabolites commonly upregulated in both species under both stress conditions; *common downregulated*—metabolites commonly downregulated in both species under both stress conditions; *heat-unique*—metabolites uniquely up- or downregulated under heat stress in both species; *cold-unique*—metabolites uniquely up- or downregulated under cold stress in both species; *NSI: heat-unique*—metabolites unique to heat stress in *N. sibirica* and neutral or not detected in *N. schoberi*; *NSI: cold-unique*—metabolites unique to cold stress in *N. sibirica* and neutral or not detected in *N. schoberi*; *NSC: heat-unique*—metabolites unique to heat stress in *N. schoberi* and neutral or not detected in *N. sibirica*; *NSC: cold-unique*—metabolites unique to cold stress in *N. schoberi* and neutral or not detected in *N. sibirica*; *species-specific*—metabolites commonly downregulated in *N. sibirica* and commonly upregulated in *N. schoberi*, or vice versa; and *stress-driven*—metabolites showing heat-unique, cold-unique, or opposite responses in one species combined with common upregulation, common downregulation, or opposite responses in the other species.

In the metabolomic profiles of *N. sibirica* and *N. schoberi* cultivated in vitro, terpenes were classified as non-stress-driven metabolites. CHDs, nitrogen-containing compounds (NCCs), PCs, and PhAEs were identified as commonly upregulated, whereas FAEs were classified as commonly downregulated (Figure 7a). FAAs and sterols were identified as heat-unique metabolites in both species, while AEEs, dioxolane Ds, MCAs, and PAEs were classified as cold-unique in *N. sibirica*. In soil-grown cultures, terpenes were identified as commonly upregulated metabolites, whereas PhAEs were classified as commonly downregulated (Figure 7b). In addition, HAADs, PAEs, and sterols were classified as heat-unique metabolites in *N. sibirica*. AAEDs, OHCCs, and ubiquinones were identified as species-specific metabolites in this cultivation system.

## 4. Discussion

Under both natural and laboratory conditions, temperature is an important physical factor influencing plant growth and development. Stress symptoms may appear within 48–72 h after temperature exposure, although this timeframe varies according to species, developmental stage, and individual sensitivity [40]. Plant responses to environmental challenges occur along a continuum from moderate stimulation to harmful impairment [26]. In the present study, exposure to +30 °C and +3 °C for 72 h was interpreted as short-term, non-lethal temperature exposure because the analysed microclones did not show visible injury and retained measurable chlorophyll fluorescence activity. But we can say about eustress for *N. sibirica* and *N. schoberi* microclones, based not solely on visible symptoms but on integrated anatomical, physiological, and metabolomic indicators. Eustress, as observed under the applied conditions, can be defined as a state in which external stimuli trigger protective and adaptive responses without exceeding thresholds that would result in irreversible damage or suppression of essential metabolic pathways. However, the distinction between eustress and distress is treated here as an operational interpretation based on the measured parameters, not as a mechanistically validated threshold.

Anatomical adaptations play a key role in supporting these functional responses by preserving structural integrity and maintaining efficient gas exchange under eustress conditions. Plant leaves are among the most sensitive organs to environmental changes, and their structural characteristics most accurately reflect plant adaptability to environmental conditions [52]. The leaves of both *N. sibirica* and *N. schoberi* are oblanceolate or oblong-scapulate, with a blunt or slightly pointed apex [28]. *Nitraria*, being a halophyte, is also a phreatophyte. The presence of a thin cuticle in *Nitraria* leaves is therefore unlikely to represent the determining structural element for maintaining water balance. Anatomical features that contribute to the survival of *Nitraria* species under arid conditions include relatively small, thick leaves with thickened leaf blades, characteristic of succulents, which were observed in both species examined. At the same time, in the leaves of *Nitraria*, a high density of trichomes and immersion of stomata into the leaf tissue were not observed. These features are not consistent with the generally accepted view that desert shrub plants possess leaves with increased hairiness, strong cutinisation, and sunken stomata to reduce water loss [53]. However, the isolateral structure and amphistomatism of *Nitraria* leaves, in which stomata are distributed on both sides of the leaf blade, ensure optimisation of transpiration and gas exchange under unfavourable temperature conditions [54,55]. Thermoregulatory adaptation of anatomical structures is further evidenced by the isopalisade organisation of the mesophyll, in which columnar parenchyma is present on both sides of the mesophyll. This feature is consistent with published data and is characteristic of xerophytic adaptation [52,56,57,58,59].

Changes in the temperature regime applied to microclones in the experiment induced species-specific adaptive modifications in tissue and organ structure, enabling the plants to maintain viability and growth under altered conditions. In particular, increases in the dimensions of protective and mechanical tissues, as well as in leaf mesophyll thickness under stress conditions, are described in the literature as structural markers of enhanced stress tolerance [7,60,61]. A pronounced reduction in stomatal width was observed in both species under both temperature treatments, representing the most consistent shared anatomical response: stomatal width decreased by 54.7–69.5% under elevated temperature and by 53.4–59.3% under low positive temperature. Such reductions in stomatal dimensions may contribute to the regulation of the balance between photosynthetic demand, gas exchange, and water loss under stress conditions [62]. In contrast, mesophyll thickness showed a species-specific response to low positive temperature, decreasing by 6.63% in *N. sibirica* but increasing by 7.42% in *N. schoberi*. These contrasting responses further support the species-specific nature of structural adjustment, with *N. schoberi* generally maintaining more stable or compensatory anatomical responses, whereas *N. sibirica* exhibited predominantly reductive changes.

Photosynthetic performance parameters provided a functional context for interpreting these structural adjustments under short-term eustress conditions. The observed mesophyll configuration forms part of a broader adaptive strategy, in which denser palisade layers combined with reduced intercellular spaces may limit internal water vapour loss while maintaining efficient light utilisation. This interpretation is consistent with the favourable *F_V_/F_M_* values observed under low positive temperature conditions, with *F_V_/F_M_* serving as a reliable indicator of the maximum photochemical efficiency of PSII [63,64]. In both *N. sibirica* and *N. schoberi*, *F_V_/F_M_* values tended to increase under low positive temperature conditions compared with the controls, suggesting maintained or potentially enhanced PSII photochemical efficiency under moderate temperature stress.

Under changing environmental conditions, plants must tightly regulate photosynthetic electron transport to prevent the accumulation of harmful reactive oxygen species [18,65,66,67]. The adverse effects of abiotic stress factors on the quantum efficiency of PSII may result from inhibition of electron transport and reaction centres at PSII sites, as well as from the onset of damage to the oxygen-evolving complex [68,69,70]. However, in the present experiment, an increase in *ETR* was observed under both high- and low-positive-temperature treatments. This finding is consistent with reports indicating that PSII-mediated electron transport may increase under low levels of stress [71] without reducing the plant’s capacity to utilise PSII energy [72]. In addition, significant positive correlations were detected between *ETR* and *F_V_/F_M_* values, with *r* = 0.51 for *N. sibirica* and *r* = 0.89 for *N. schoberi*. Under high-temperature treatment, *N. sibirica* showed a slight, non-significant downward trend in *F_V_/F_M_* accompanied by a significant increase in *Y*(*NO*), indicating enhanced non-regulated energy dissipation [73]. At the same time, the significant increase in *Y*(*NPQ*) indicates the simultaneous activation of regulated photoprotective energy dissipation. Thus, although the increase in *Y*(*NO*) suggests greater excitation pressure on the photosynthetic apparatus, the concurrent enhancement of *Y*(*NPQ*), together with the absence of a significant decline in *F_V_/F_M_*, indicates that photoprotective regulation remained functionally effective under the applied high-temperature conditions [74,75,76]. Such activation of regulated energy dissipation is consistent with a eustress response, allowing the plant to accommodate increased excitation pressure while limiting the risk of photoinhibitory and oxidative damage [77,78].

Metabolomic profiling can extend the analysis beyond observable structural traits and photosynthetic performance. But given that the present findings are based on a single experiment, broader theoretical conclusions would require further experimental validation. The obtained GC–MS results indicate treatment-associated shifts in the relative abundance and diversity of putatively annotated compound classes. Crucially, the observed changes should be interpreted in terms of relative TIC-normalised GC–MS profiles rather than absolute metabolite concentrations, and terms such as “increase,” “decrease,” or “expansion” refer to relative representation or detectability within these profiles, not to validated changes in absolute metabolite accumulation. This distinction is important because peak-area normalisation describes compositional patterns and can be influenced by changes in other peaks within the chromatogram [47,48,50].

At the same time, the observed changes across anatomical, physiological, and metabolomic traits provide a basis for considering the emerging response patterns and their possible biological significance. It is generally accepted that temperature-induced damage in plants begins with a rapid increase in membrane rigidity, resulting from phase transitions within the lipid bilayer [79]. Fatty acids and their derivatives detected in the metabolomic profiles of both *N. sibirica* and *N. schoberi* following high- and low-temperature exposure are typically localised within the lipid components of chloroplast and mitochondrial membranes, and increased levels of fatty acid derivatives may contribute to attenuation of PSII photoinhibition [80]. Fatty acids also serve as precursors of major phytohormones, such as jasmonates, which are actively involved in stress adaptation processes [18,80,81]. The identified phosphoric acid esters indicate that, when primary photosynthetic products are utilised for respiration, energy stored in high-energy phosphoric acid ester bonds is channelled into growth processes to maintain the current physiological state.

In microclones of both *Nitraria* species, active biosynthesis of terpene compounds was observed under both control and stress conditions. These compounds play a key role in lipid metabolism and membrane organisation, contributing to enhanced interactions between membrane proteins and lipid components, thereby stabilising cellular membranes under stress conditions [82,83]. Environmental influences on mesophyll structure and organisation may also affect chlorophyll turnover. Under such conditions, chlorophyll degradation releases phytol, which is subsequently metabolised. The breakdown of phytol promotes the synthesis of tocopherols (vitamin E) and other protective metabolites, contributing to antioxidant defence and membrane stability [84,85]. In turn, phenols and flavonoids are involved in photosynthesis, suppression or scavenging of free radicals, modulation of respiration and oxidative phosphorylation, and alteration of membrane permeability and, as a rule, exert cytoprotective effects [86,87,88]. In addition, plants utilise these phenolic compounds containing carboxyl groups on the benzene ring to scavenge free radicals, form protective conjugates (e.g., sugars bound to the benzene ring), and modulate transpiration [89,90].

We propose that the synthesis of phenolic compounds in *Nitraria* microclones may be associated with temperature-induced changes in the thickness of both abaxial and adaxial epidermal layers during heat treatment [91,92]. In *N. sibirica*, such structural reinforcement was correlated with the deposition of phenolic compounds within cell walls, indicating the integration of biochemical defence mechanisms into the anatomical framework. In contrast, *N. schoberi* maintained relatively stable tissue dimensions across treatments, suggesting the presence of an inherent resilience strategy that relies less on morphological reconfiguration. The absence of pronounced reductions in key metabolite classes, such as terpenes and organoheterocyclic compounds, further indicates that overall metabolic integrity was preserved under the applied eustress conditions. In contrast, distress would be expected to manifest as a broad depletion of metabolites and disruption of essential biosynthetic pathways.

Interestingly, sensitivity patterns differed between the two species. *N. schoberi* exhibited greater stability across temperature treatments, with more uniform responses and enhanced non-enzymatic defence efficiency. In contrast, *N. sibirica* displayed more pronounced variability: its metabolomic repertoire expanded substantially under low-temperature in vitro conditions but showed greater fluctuations under elevated temperatures at both physiological and biochemical levels. This divergence suggests species-specific thresholds for the transition from eustress to distress.

The relatively stable anatomical profile of *N. schoberi* across control and temperature treatments indicates an inherent structural resilience, likely shaped by its evolutionary adaptation [28]. This stability may allow metabolic resources to be preferentially allocated towards biochemical defence mechanisms rather than continuous morphological adjustment. Furthermore, the apparent capacity of *N. schoberi* to sustain non-enzymatic antioxidant systems more effectively than *N. sibirica* may be linked to its broader ecological amplitude and distribution patterns [34,93,94]. This pattern is reflected in the present dataset, where terpene levels remained stable across treatments, while phenolic profiles adjusted proportionally without disrupting core metabolic pathways. Such characteristics suggest that selection has favoured a strategy combining biochemical flexibility with structural stability, thereby reducing vulnerability to rapid climatic fluctuations. Conversely, *N. sibirica*, although also stress-tolerant, exhibited substantial shifts in metabolomic diversity under low-temperature in vitro conditions, with an expansion from four to thirteen classes of compounds. From an evolutionary perspective, such plasticity may confer advantages during episodic favourable periods following stress, enabling rapid synthesis of secondary metabolites involved in defence and signalling [95]. However, the slight downward tendency in *F_V_/F_M_* observed in *N. sibirica* under elevated temperature, despite the absence of a corresponding increase in *Y*(*NO*), may indicate a somewhat greater physiological sensitivity to this treatment compared with *N. schoberi*. The persistence of these adaptive strategies is consistent with the survival requirements of perennial shrubs inhabiting desert and arid ecosystems. Further experiments across a broader temperature range would be required to determine whether this tendency reflects differences in upper thermal tolerance between the species.

The integration of anatomical stability with biochemical responsiveness may reflect a common adaptive framework shaped by similar environmental pressures, including water limitation, excess radiation, and oxidative stress [28]. At the same time, species-specific ecological and evolutionary histories may contribute to the observed interspecific differences in response patterns.

Environmental conditions during growth determine how evolutionarily developed adaptive capacities are expressed under experimental settings [96,97]. In vitro microclones, maintained under controlled conditions with reduced environmental heterogeneity and without interactions with soil microbial communities, exhibited high biosynthetic potential when exposed to stimulatory conditions [98]. In contrast, soil-adapted microclones responded more conservatively: metabolomic diversity generally decreased under both high- and low-temperature treatments relative to control conditions while maintaining compositionally balanced profiles enriched in essential protective compounds, such as phenolics, except where selectively reduced by specific treatments. The reduction or absence of certain metabolite classes, for example, the loss of flavonoids under high-temperature soil cultivation, may reflect selective metabolic reprioritisation rather than systemic metabolic disruption, particularly given the continued presence of other defensive compounds [99,100]. This pattern may reflect an ecological trade-off, whereby acclimation to a complex growth environment favours maintenance of a functionally balanced metabolomic profile rather than expansion of metabolomic diversity. Soil-grown plants develop within complex microhabitats, where interactions among roots, edaphic factors, and soil microbial communities contribute to shaping plant metabolic responses [101,102]. As a result, metabolomic responses remain conservative yet functionally effective.

The observed differences between growth systems (in vitro versus soil cultivation) illustrate how environmental complexity influences whether plant responses manifest as increased metabolomic diversity or compositional stability while still maintaining the defining characteristics that distinguish eustress from distress. These findings highlight that plant adaptation is not uniform but is intricately modulated by both the nature of the stressor and the growth environment [100].

This complexity underscores the need for integrative studies addressing the multicomponent nature of plant adaptation. Taken together, microclone-based experiments in *N. sibirica* and *N. schoberi* revealed treatment-dependent metabolic diversification, maintenance or enhancement of photoprotective performance, and structural plasticity, collectively supporting the interpretation of the applied temperature treatments as eustress rather than distress [96,100]. The distinction between these two states becomes evident only when considering these factors collectively rather than in isolation. Anatomical, physiological, and metabolic processes interact functionally [103]. The integration of multiple data streams demonstrates how the interplay between metabolic reprogramming, photosynthetic regulation, and anatomical adaptation determines whether stress acts as beneficial stimulation or harmful disruption. But the presented design does not allow these alternatives to be separated mechanistically. Therefore, the contrast between in vitro and soil-acclimatised plants should be interpreted as a cultivation-system-associated pattern requiring further testing.

The combined anatomical, photosynthetic, and metabolomic datasets indicate that the responses of *N. sibirica* and *N. schoberi* to short-term temperature exposure were multidirectional rather than uniformly coordinated. In some cases, physiological stability occurred together with marked shifts in relative metabolite profiles, whereas anatomical changes did not always correspond directly to fluorescence responses or metabolomic diversity. For example, maintenance of *F_V_/F_M_* and *ETR* under some treatments suggests that severe impairment of PSII was not evident, but this does not demonstrate direct activation of specific biosynthetic pathways. Similarly, changes in stomatal dimensions, mesophyll thickness, or vascular bundle diameter may reflect anatomical adjustment to temperature exposure, but they cannot be interpreted as direct drivers of metabolite accumulation without additional mechanistic experiments. Therefore, the relationships between anatomical structure, photosynthetic regulation, and metabolomic composition should be interpreted as treatment-associated patterns rather than established causal mechanisms.

The majority of putatively annotated metabolites and metabolite classes detected in *N. sibirica* and *N. schoberi* have been previously reported in the literature as compounds with biological or pharmacological relevance. However, the present study did not experimentally assess antioxidant, antimicrobial, anti-inflammatory, anticancer, therapeutic, or nutraceutical activity. Therefore, these literature-derived associations should be interpreted as hypotheses for future targeted validation rather than as experimentally confirmed properties of the detected metabolites [79,104,105] (Table S9).

Thus, taken together, all these observations indicate that short-term, non-lethal temperature treatments were associated with changes in the relative composition and diversity of putatively annotated metabolite classes in *Nitraria* microclones. Photochemical indicators alone may suggest physiological strain under elevated temperatures; however, when interpreted alongside enhanced regulated energy dissipation (increased *Y*(*NPQ*) without a corresponding increase in *Y*(*NO*)), reinforced anatomical traits, and metabolomic profiles rich in compounds such as flavonoids and phenolics, the integrated response indicates that the microclones maintained functional resilience without showing signs of transition from eustress to distress.

Overall, this comparative framework demonstrates that controlled thermal modulation in laboratory conditions can activate adaptive reactions of plants without compromising essential physiological functions. This observation supports the concept that mild abiotic stress could in the future be strategically applied to stimulate targeted biosynthetic pathways in halophytic medicinal plants while avoiding detrimental effects.

At the same time, the present study provides only a descriptive framework for comparing temperature-associated anatomical, photosynthetic, and relative GC–MS profile responses in *Nitraria* microclones. It identifies response patterns and candidate compound classes for future validation, but it does not establish causal links among response levels or confirm biological activity. Future work should include replicate-level chromatographic reporting, authentic standards, retention-index confirmation, absolute quantification, technical injection replicates, paired multi-omics sampling, and functional bioactivity assays to determine whether the observed relative GC–MS patterns correspond to confirmed biochemical or biological functions.

## 5. Conclusions

The present study demonstrated that short-term exposure of *Nitraria sibirica* and *Nitraria schoberi* microclones to elevated (+30 °C) and low positive (+3 °C) temperatures induced coordinated but species- and cultivation-system-dependent anatomical, photosynthetic, and relative metabolomic responses without clear disruption of key physiological processes. Overall, the observed variation was consistent with short-term eustress responses under the applied experimental conditions.

The key differences were evident at several levels. *N. schoberi* generally demonstrated more stable or compensatory responses across the analysed traits, particularly under low positive temperature exposure. At the photosynthetic level, high-temperature exposure increased *Y*(*NPQ*) 3.33-fold in *N. sibirica* and 4.07-fold in *N. schoberi*, indicating pronounced activation of regulated non-photochemical energy dissipation. Metabolomic responses were particularly pronounced in *N. sibirica* under low-temperature in vitro conditions, where the number of detected putative compound classes increased from 4 to 13. More broadly, in vitro temperature treatments were associated with greater diversification of relative metabolomic profiles, whereas soil-acclimatised plants exhibited comparatively greater compositional stability across temperature regimes.

The integrated dataset therefore supports the use of *Nitraria* microclones as an experimental system for studying temperature-associated structural, photosynthetic, and relative metabolomic responses in halophytic species. At the same time, the observed relationships among these response levels represent treatment-associated patterns rather than established causal mechanisms, and the present GC–MS data describe relative profiles of putatively annotated compounds rather than validated metabolic pathways or absolute metabolite concentrations.

Future studies should combine targeted chemical validation using authentic standards, absolute quantification, orthogonal analytical methods, and functional bioactivity assays to confirm metabolite identities and determine whether the observed temperature-associated metabolomic responses have biological activity and potential practical value.

## Figures and Tables

**Table 1:** Adaptive changes in leaf anatomical parameters of *N. sibirica* and *N. schoberi* microclones in vitro. Different letters indicate significant difference within the one parameter of species between treatments (conditions).

Conditions	Adaxial Epidermis, µm		Abaxial Epidermis, µm		Mesophyll, µm	
	*N. sibirica*	*N. schoberi*	*N. sibirica*	*N. schoberi*	*N. sibirica*	*N. schoberi*
Ctrl	24.4 ± 0.32 b	25.3 ± 0.18	24.5 ± 0.19 b	25.6 ± 0.21 b	362 ± 2.23 a	364 ± 2.35 b
30 °C	25.5 ± 0.24 a	25.2 ± 0.18	25.4 ± 0.17 a	23.9 ± 0.28 c	362 ± 2.08 a	367 ± 1.12 b
3 °C	23.6 ± 0.22 c	25.4 ± 0.37	24.1 ± 0.18 c	27.4 ± 0.21 a	338 ± 2.01 b	391 ± 5.93 a
*p*-value	<0.001	0.654	<0.001	<0.001	<0.001	<0.001
**Conditions**	**Central Bundle D, µm**		**Stomata Length, µm**		**Stomata Width, µm**	
	***N. sibirica***	***N. schoberi***	***N. sibirica***	***N. schoberi***	***N. sibirica***	***N. schoberi***
Ctrl	140 ± 0.75 a	117 ± 0.64 b	123 ± 0.92 a	108 ± 0.38 c	25.3 ± 0.17 a	23.6 ± 0.18 a
30 °C	128 ± 1.02 b	102 ± 0.74 c	110 ± 0.56 b	113 ± 0.48 b	10.0 ± 0.15 b	10.7 ± 0.22 b
3 °C	130 ± 0.80 b	120 ± 2.10 a	107 ± 0.49 c	117 ± 0.99 a	10.3 ± 0.17 b	11.0 ± 0.48 b
*p*-value	<0.001	<0.001	<0.001	<0.001	<0.001	<0.001

## Data Availability

All used data is presented within the manuscript and Supplementary Materials.

## Supplementary Materials

The following supporting information can be downloaded at https://www.mdpi.com/article/10.3390/metabo16090646/s1, Figure S1: GC–MS total ion chromatograms of ethanol extracts from *N. sibirica* and *N. schoberi* microclones: (a) control; (b) elevated temperature (+30 °C, 72 h); (c) low positive temperature (+3 °C, 72 h) treatments in vitro; Figure S2: GC–MS total ion chromatograms of ethanol extracts from soil-grown *N. sibirica* and *N. schoberi* microclones: (a) control; (b) elevated temperature (+30 °C, 72 h); (c) low positive temperature (+3 °C, 72 h) treatments; Table S1: Traceability; Table S2: Detected peaks only; Table S3: Evidence level; Table S4: Adaptive changes in photosynthetic activities in leaves of *N. sibirica* and *N. schoberi* microclones in vitro; Table S5: Adaptive changes in metabolomic profile of *N. sibirica* microclones grown under in vitro conditions; Table S6: Adaptive changes in metabolomic profile of *N. schoberi* microclones grown under in vitro conditions; Table S7: Adaptive changes in metabolomic profile of soil-grown *N. sibirica*; Table S8: Adaptive changes in metabolomic profile of soil-grown *N. schoberi*; Table S9: Biologically active properties of metabolites identified in microclones of *N. sibirica* and *N. schoberi* under stress conditions.

## Abbreviations

The following abbreviations are used in this manuscript:

AAEDs	Alcohols, aldehydes, ethers, and their derivatives
AEEs	Aspirates, esters, and ethers
AHCs	Alkane hydrocarbons
ANOVA	Analysis of variance
BACs	Biologically active compounds
BAP	6-Benzylaminopurine
CADs	Carboxylic acids and their derivatives
CHDs	Carbohydrates and their derivatives
DCDB	Dicarboxylic derivatives of benzene
DFFP	Derivatives of furan, furanone, and pyran
Dioxolane Ds	Dioxolane derivatives
ETR	Electron transport rate
FAAs	Fatty acid amides
FAEs	Fatty acids and their esters
GC–MS	Gas chromatography–mass spectrometry
HAADs	Hydroxyamino acid derivatives
IBA	Indole-3-butyric acid
LC–MS	Liquid chromatography–mass spectrometry
MCAs	Monocarboxylic acids
MS/MS	Tandem mass spectrometry
*m/z*	Mass-to-charge ratio
NCCs	Nitrogen-containing compounds
OHCCs	Organoheterocyclic compounds
PAEs	Phosphoric acid esters
PAM	Pulse-amplitude modulation
PCs	Phenolic compounds
PhAEs	Phthalic acid esters
PSII	Photosystem II
RLCs	Rapid light curves
TIC	Total ion chromatogram
