# Supplementary material for: Temperature-Associated Anatomical, Photosynthetic, and Putative GC–MS Metabolomic Profiles in the Arid Species Nitraria sibirica Pall. and Nitraria schoberi L
Source: Metabolites. 2026 Sep 3;16(9):646. doi: 10.3390/metabo16090646 (PMC13609096; doi:10.3390/metabo16090646)
Supplement: Supplementary file 1 [file metabolites-16-00646-s001.zip › 20260715 Supplementary File_S2.pdf]

N. sibirica ***N. schoberi***

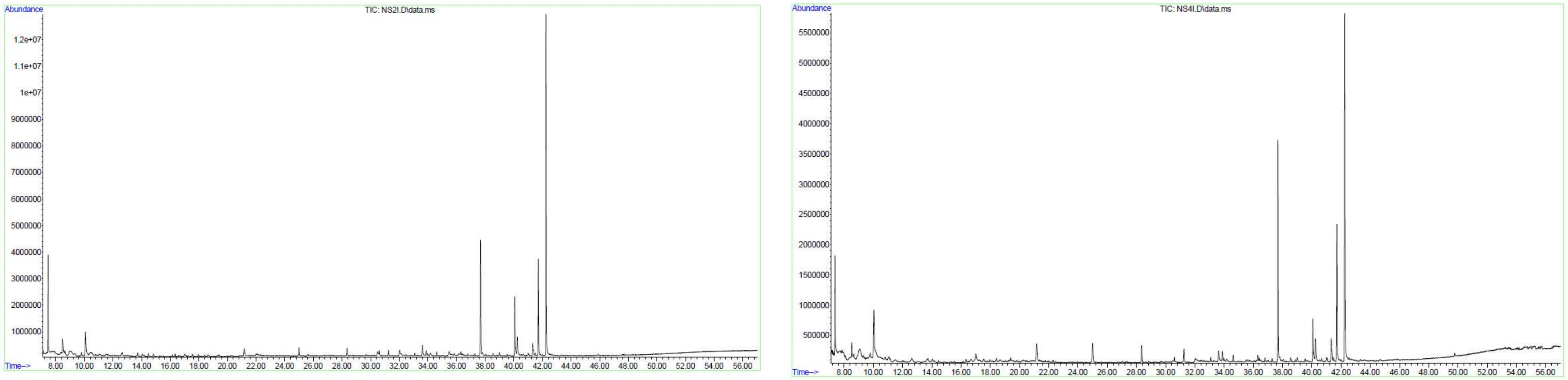

a

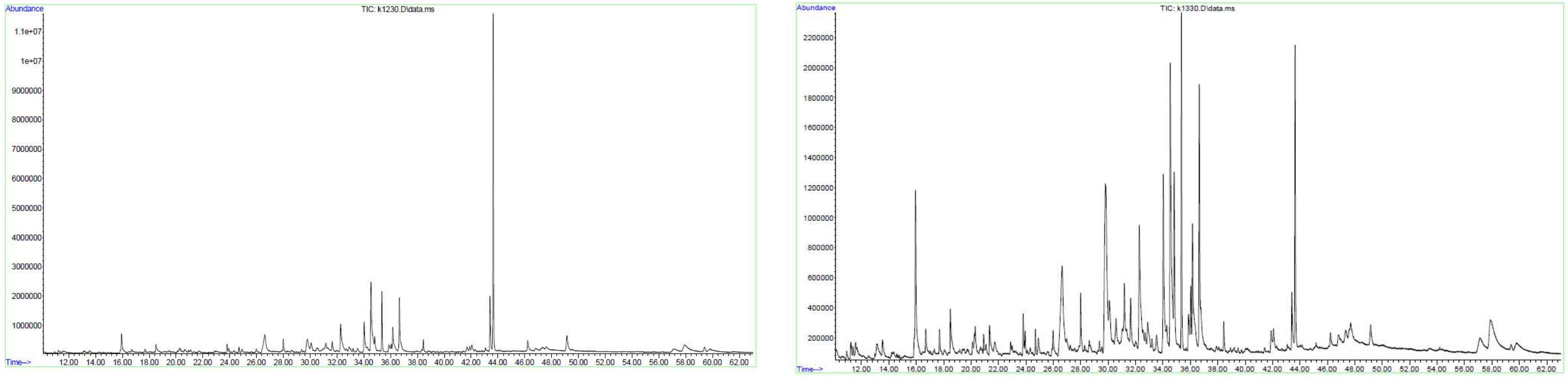

b

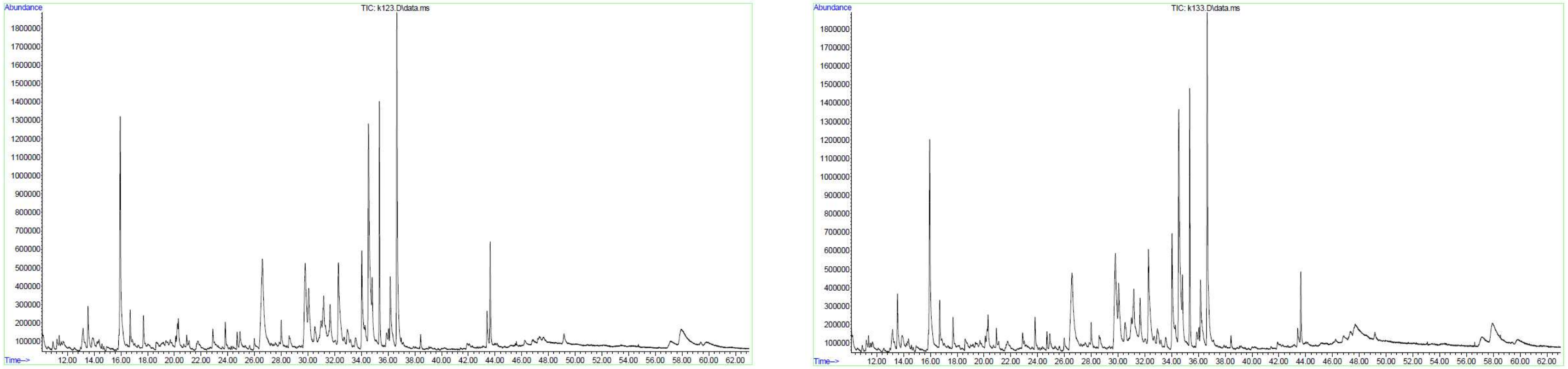

c

Figure S1. GC–MS total ion chromatograms of ethanol extracts from *N. sibirica* and *N. schoberi* microclones: a) control; b) elevated temperature (+30 °C, 72 h); c) low positive temperature (+3 °C, 72 h) treatments *in vitro*.

N. sibirica *N. schoberi*

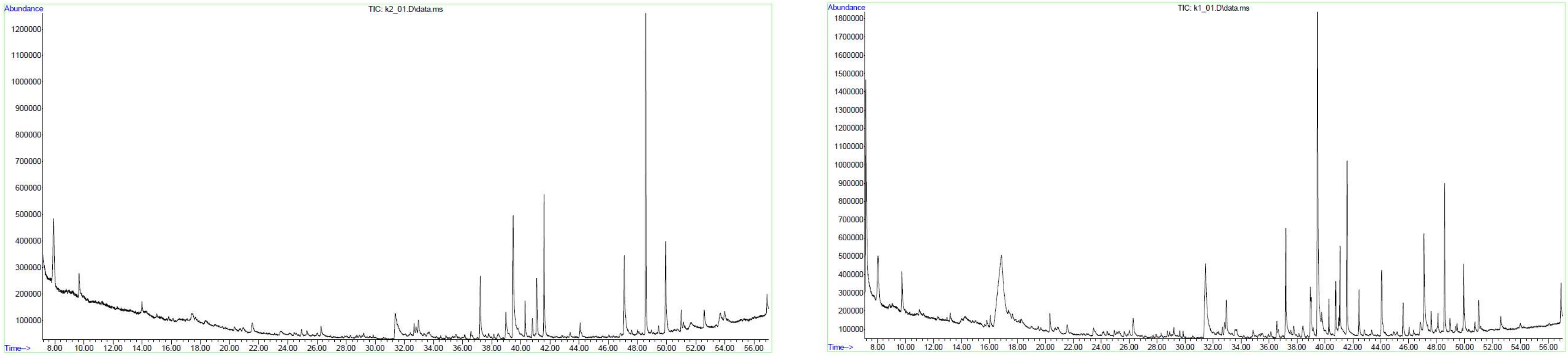

a

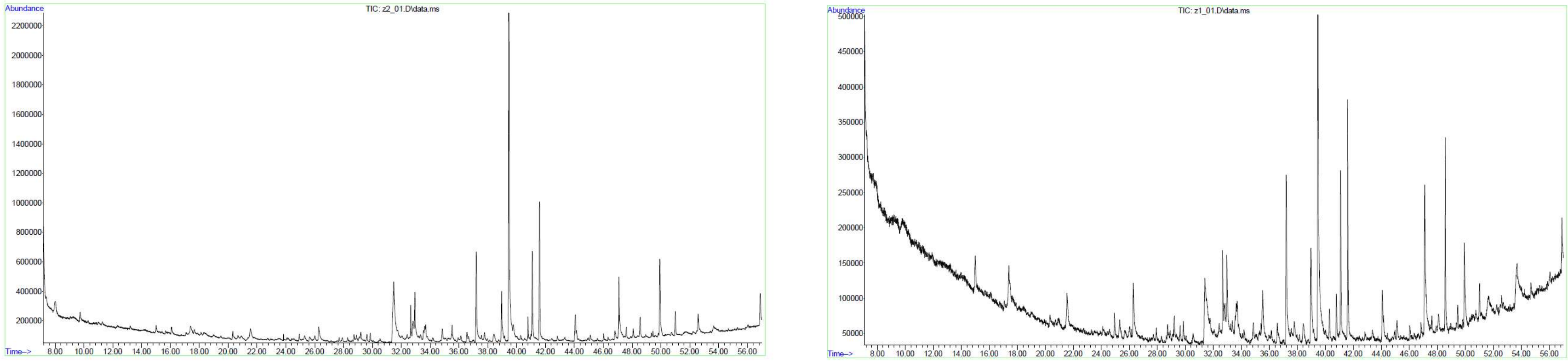

b

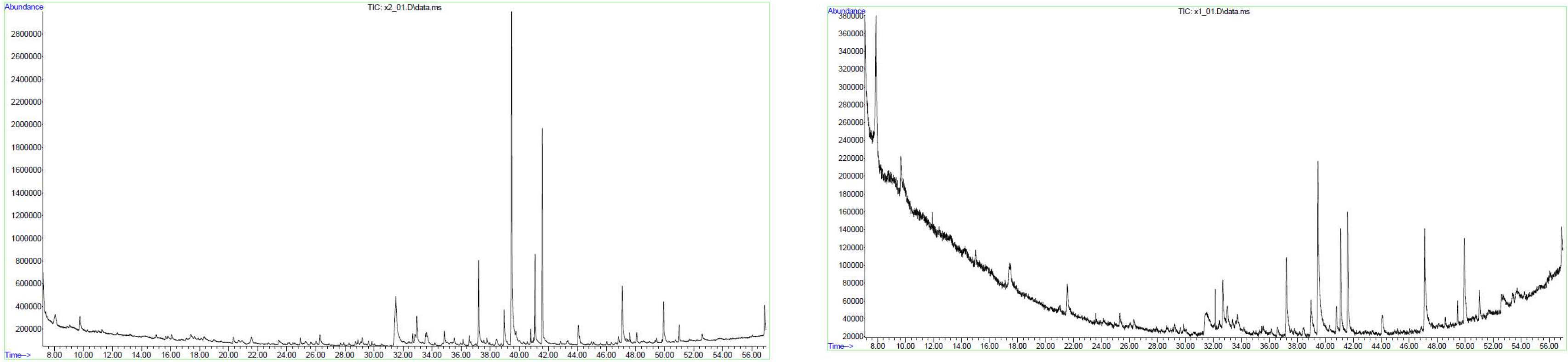

c

Figure S2. GC–MS total ion chromatograms of ethanol extracts from soil-grown *N. sibirica* and *N. schoberi* microclones: a) control; b) elevated temperature (+30 °C, 72 h); c) low positive temperature (+3 °C, 72 h) treatments.

Table S4. Adaptive changes in photosynthetic activities in leaves of *N. sibirica* and *N. schoberi* microclones *in vitro*.

Conditions	F_V/F_M		ETR_{65}	
	N. sibirica	*N. schoberi*	*N. sibirica*	*N. schoberi*
Control	0.667 ± 0.005 [b]	0.710 ± 0.017 [b]	19.3 ± 1.16 [b]	17.5 ± 0.38
"Heat"	0.641 ± 0.013 [b]	0.690 ± 0.047 [b]	18.8 ± 1.10 [b]	18.6 ± 2.50
"Cold"	0.784 ± 0.024 [a]	0.795 ± 0.009 [a]	23.2 ± 2.15 [a]	19.8 ± 1.58
p-value	< 0.001	< 0.01	< 0.05	0.188

Conditions	$Y(II)_{625}$		$Y(NO)_{65}$		$Y(NPQ)_{65}$	
	N. sibirica	*N. schoberi*	*N. sibirica*	*N. schoberi*	*N. sibirica*	*N. schoberi*
Control	0.257 ± 0.033 [b]	0.286 ± 0.016 [ab]	0.366 ± 0.015 [b]	0.446 ± 0.017 [a]	0.030 ± 0.006 [b]	0.029 ± 0.005 [b]
"Heat"	0.488 ± 0.021 [a]	0.356 ± 0.006 [a]	0.448 ± 0.015 [a]	0.297 ± 0.014 [c]	0.100 ± 0 [a]	0.118 ± 0.005 [a]
"Cold"	0.296 ± 0.030 [b]	0.212 ± 0.054 [b]	0.375 ± 0.015 [b]	0.357 ± 0.015 [b]	0.034 ± 0.005 [b]	0.013 ± 0.002 [c]
p-value	< 0.001	< 0.01	< 0.001	< 0.001	< 0.001	< 0.001

Table S5. Adaptive changes in metabolomic profile of *N. sibirica* microclones grown under *in vitro* conditions.

Compound	Control			30 °C			3 °C		
	Content, %	RT, min	IP, %	Content, %	RT, min	IP, %	Content, %	RT, min	IP, %
**Monocarboxylic acids [MCA]**									
Butanoic acid, 4-hydroxy-	–	–	–	–	–	–	0.37	11.213	86
**SUM**	–	–	–	–	–	–	**0.37**	–	–
**Fatty acids and their esters [FAE]**									
9,12,15-Octadecatrienoic acid, (*Z,Z,Z*)-	–	–	–	1.70	36.754	80	–	–	–
Hexadecanoic acid, ethyl ester (palmitic acid ester)	14.24	32.247	91	6.01	32.273	85	6.31	32.281	87
Octadecanoic acid, ethyl ester	1.75	35.830	76	1.31	35.863	73	–	–	–
9,12-Octadecadienoic acid, ethyl ester (linoleic acid ester)	11.21	36.131	91	5.07	36.155	88	4.18	36.156	87
9,12,15-Octadecatrienoic acid, ethyl ester, (*Z,Z,Z*)-	40.91	36.633	95	6.36	36.653	91	13.81	36.654	93
**SUM**	**68.11**	–	–	**20.45**	–	–	**24.30**	–	–
**Fatty acid amides [FAA]**									
9-Octadecenamide, (*Z*)-	–	–	–	0.93	41.694	73	–	–	–
13-Docosenamide, (*Z*)-	–	–	–	–	–	–	–	–	–
**SUM**	–	–	–	**0.93**	–	–	–	–	–
**Phosphoric acid esters [PAE]**									
Phosphoric acid, diethyl octyl ester	–	–	–	–	–	–	0.69	24.912	68
**SUM**	–	–	–	–	–	–	**0.69**	–	–
**Carbohydrates and their derivatives [CHD]**									
Sucrose	–	–	–	7.13	26.615	71	10.82	26.585	72
Ethyl *α-D*-glucopyranoside	7.66	29.751	83	4.05	29.787	80	6.84	29.793	81
**SUM**	**7.66**	–	–	**11.18**	–	–	**17.66**	–	–
**Nitrogen-containing compounds [NCC]**									

2-Propanamine, *N*-methyl-*N*-nitroso-	–	–	–	–	–	–	0.94	17.689	71
3-Dodecyne, 2-amino-2-methyl-	–	–	–	0.54	24.693	73	0.50	24.703	71
1,3-Dicyclohexylurea	–	–	–	1.42	38.439	85	0.48	38.429	75
**SUM**	–	–	–	**1.96**	–	–	**1.92**	–	–
**Alcohols, esters, and ethers [AEE]**									
Cyclopropyl carbinol	–	–	–	–	–	–	1.05	16.693	77
Allyl acetate	–	–	–	–	–	–	1.04	20.304	70
Ethanol, 2-(2-butoxyethoxy)-, acetate	–	–	–	–	–	–	0.28	20.921	76
**SUM**	–	–	–	–	–	–	**2.37**	–	–
**Derivatives of furan, furanone, and pyran [DFFP]**									
2-Furancarboxaldehyde, 5-methyl-	–	–	–	–	–	–	0.39	10.915	80
2(5H)-Furanone	–	–	–	–	–	–	0.29	11.380	78
2-Hydroxy-gamma-butyrolactone	–	–	–	–	–	–	1.87	13.536	87
4H-Pyran-4-one, 2,3-dihydro-3,5-dihydroxy-6-methyl-	–	–	–	3.67	15.936	89	11.32	15.949	91
Maltol	–	–	–	–	–	–	0.38	13.940	67
**SUM**	–	–	–	**3.67**	–	–	**14.25**	–	–
**Phenolic compounds [PC]**									
Acetamide, N-(4-ethoxy-3-hydroxyphenyl)-	–	–	–	1.07	36.028	66	0.69	36.031	64
Phenol, 2,2'-methylenebis[6-(1,1-dimethylethyl)-4-methyl-	–	–	–	29.88	43.650	93	4.45	43.640	90
3',5'-Dimethoxyacetophenone	–	–	–	1.54	27.996	76	–	–	–
**SUM**	–	–	–	**32.49**	–	–	**5.14**	–	–
**Flavonoids**									
2H-1-Benzopyran-3,4-diol, 2-(3,4-dimethoxyphenyl)-3,4-dihydro-6-methyl-, (2α,3α,4α)-	–	–	–	–	–	–	1.17	27.998	78
**SUM**	–	–	–	–	–	–	**1.17**	–	–

**Organoheterocyclic compounds [OHCC]**									
2,9-Dimethyldecahydroquinol-4-one	–	–	–	–	–	–	0.78	23.815	72
1-Acetyl-2-methyl-6-(prop-2-enyl)piperidine (*N*-Acetylpinidine)	0.96	23.79	72	0.78	23.805	74	–	–	–
2-Pyridineacetic acid, hexahydro-	–	–	–	1.76	18.495	80	–	–	–
Dodecahydro-7a,11b-diaza-benzo[de]anthracene	8.48	34.002	86	3.55	34.026	86	4.80	34.030	85
Oxazole, 5-hexyl-2,4-dimethyl	–	–	–	2.11	34.809	69	3.92	34.804	68
**SUM**	**9.44**	–	–	**8.20**	–	–	**9.50**	–	–
**Sterols**									
Stigmasterol	–	–	–	2.89	57.913	76	–	–	–
**SUM**	–	–	–	**2.89**	–	–	–	–	–
**Phthalic acid esters [PhAE]**									
Dibutyl phthalate	–	–	–	5.30	35.350	96	6.63	35.348	96
**SUM**	–	–	–	**5.30**	–	–	**6.63**	–	–
**Dioxolane derivative [Dioxolane D]**									
1,3-Dioxol-2-one,4,5-dimethyl-	–	–	–	–	–	–	1.43	13.170	66
**SUM**	–	–	–	–	–	–	**1.43**	–	–
**Terpenes**									
Phytol	14.79	34.504	94	12.93	34.531	93	14.57	34.530	92
**SUM**	**14.79**	–	–	**12.93**	–	–	**14.57**	–	–

Notes: RT – retention time; IP – identification probability.

Table S6. Adaptive changes in metabolomic profile of *N. schoberi* microclones grown under *in vitro* conditions.

Compound	Control			30 °C			3 °C		
	**Content, %**	**RT, min**	**IP, %**	**Content, %**	**RT, min**	**IP, %**	**Content, %**	**RT, min**	**IP, %**
**Fatty acids and their esters [FAE]**									
9,12,15-Octadecatrienoic acid, (Z,Z,Z)-	–	–	–	1.63	36.765	79	–	–	–
Hexadecanoic acid, ethyl ester (palmitic acid ester)	16.62	32.250	92	5.71	32.277	88	7.24	32.272	87
Octadecanoic acid, ethyl ester	1.95	35.834	76	1.31	35.864	72	–	–	–
9,12-Octadecadienoic acid, ethyl ester (linoleic acid ester)	15.16	36.129	92	5.34	36.155	88	3.94	36.157	86
9,12,15-Octadecatrienoic acid, ethyl ester, (*Z,Z,Z*)-	46.28	36.632	95	6.03	36.652	89	13.00	36.652	93
**SUM**	**80.01**	–	–	**20.02**	–	–	**24.18**	–	–
**Phosphoric acid esters [PAE]**									
Phosphoric acid, diethyl octyl ester	–	–	–	0.58	24.907	68	–	–	–
**SUM**	–	–	–	**0.58**	–	–	–	–	–
**Carbohydrates and their derivatives [CHD]**									
Sucrose	–	–	–	8.05	26.643	76	9.48	26.566	75
α-D-Glucopyranoside, methyl	–	–	–	11.47	29.802	79	–	–	–
Ethyl *α-D*-glucopyranoside	–	–	–	2.17	30,088	77	7.87	29.802	85
**SUM**	–	–	–	**21.69**	–	–	**17.35**	–	–
**Nitrogen-containing compounds [NCC]**									
2-Propanamine, N-methyl-N-nitroso-	–	–	–	0.59	17.695	71	1.19	17.688	72
3-Dodecyne, 2-amino-2-methyl-	–	–	–	–	–	–	0.54	24.699	72
2-Pyrrolidinecarboxylic acid-5-oxo-, ethyl ester	–	–	–	0.89	25.987	81	–	–	–
1,3-Dicyclohexylurea	–	–	–	0.65	38.438	80	0.44	38.431	76
**SUM**	–	–	–	**2.13**	–	–	**2.17**	–	–

**Alcohols, aldehydes, esters and their derivatives [AAED]**									
1,4-Butanediol, diacetate	–	–	–	–	–	–	0.44	14.364	67
Cyclopropyl carbinol	–	–	–	0.91	16.694	75	1.51	16.694	77
Ethanol, 2-(2-butoxyethoxy)-, acetate	–	–	–	0.49	20.918	86	0.43	20.922	82
(Hexahydropyrrolizin-3-ylidene)-acetaldehyde	–	–	–	0.52	23.945	76	–	–	–
**SUM**	–	–	–	**1.92**	–	–	**2.38**	–	–
**Derivatives of furan, furanone, and pyran [DFFP]**									
Butyrolactone	–	–	–	0.47	11.234	74	0.46	11.220	80
2(5H)-Furanone	–	–	–	0.23	11.388	76	0.47	11.381	75
2,5-Dimethyl-4-hydroxy-3(2H)-furanone	–	–	–	0.73	13.113	75	–	–	–
2-Hydroxy-gamma-butyrolactone	–	–	–	0.78	13.539	76	2.46	13.545	85
4H-Pyran-4-one, 2,3-dihydro-3,5-dihydroxy-6-methyl-	–	–	–	6.05	15.947	91	10.50	15.949	91
5-Hydroxymethyldihydrofuran-2-one	–	–	–	0.33	20.109	77	0.36	20.102	75
Maltol	–	–	–	–	–	–	1.44	13.895	69
**SUM**	–	–	–	**8.59**	–	–	**15.69**	–	–
**Phenolic compounds [PC]**									
Phenol, 2,6-dimethoxy-	–	–	–	0.19	22.882	69	–	–	–
Phenol, 3,5-bis(1-pyrrolidinyl)-	–	–	–	0.70	33.534	68	–	–	–
Acetamide, N-(4-ethoxy-3-hydroxyphenyl)-	–	–	–	1.61	36.031	71	–	–	–
Phenol, 2,2'-methylenebis[6-(1,1-dimethylethyl)-4-methyl-	–	–	–	6.25	43.641	92	2.31	43.639	87
**SUM**	–	–	–	**8.75**	–	–	**2.31**	–	–
**Flavonoids**									
2H-1-Benzopyran-3,4-diol, 2-(3,4-dimethoxyphenyl)-3,4-dihydro-6-methyl-, (2α,3α,4α)-	–	–	–	0.91	26.987	78	0.89	27.995	72

2-(3,4-Dimethoxyphenyl)-6-methyl-3,4-chromanediol	1.59	27.985	77	–	–	–	–	–	–
**SUM**	**1.59**	–	–	**0.91**	–	–	**0.89**	–	–
**Organoheterocyclic compounds [OHCC]**									
2-Pyrrolidinone, 5-(cyclohexylmethyl)-	–	–	–	–	–	–	0.75	25.986	74
2-Methyl-9-β-d-ribofuranosylhypoxanthine	–	–	–	–	–	–	5.30	31.180	66
Dodecahydro-7a,11b-diaza-benzo[de]anthracene	6.39	34.004	85	5.53	34.025	85	5.40	34.029	85
Oxazole, 5-hexyl-2,4-dimethyl	–	–	–	5.43	34.818	71	3.18	34.805	69
2-Pyridineacetic acid, hexahydro-	–	–	–	1.66	18.490	80	–	–	–
1-Acetyl-2-methyl-6-(prop-2-enyl)piperidine (N-Acetylpinidine)	–	–	–	0.94	23.803	74	0.91	23.810	71
Pyrazine, 2-ethyl-5-methyl-	–	–	–	1.19	21.348	79	–	–	–
1-(1'-pyrrolidinyl)-2-propanone	–	–	–	0.18	11.573	85	–	–	–
**SUM**	**6.39**	–	–	**14.93**	–	–	**15.54**	–	–
**Sterols**									
Stigmasterol	–	–	–	2.67	57.889	69	–	–	–
**SUM**	–	–	–	**2.67**	–	–	–	–	–
**Phthalic acid esters [PhAE]**									
Dibutyl phthalate	–	–	–	6.36	35.348	95	6.22	35.348	95
**SUM**	–	–	–	**6.36**	–	–	**6.22**	–	–
**Terpenes**									
Phytol	12.02	34.508	93	11.45	34.533	94	13.27	34.533	92
**SUM**	**12.02**	–	–	**11.45**	–	–	**13.27**	–	–

Notes: RT – retention time; IP – identification probability.

Table S7. Adaptive changes in metabolomic profile of soil-grown *N. sibirica.*

Compound	Control			30 °C			+3 °C		
	Content, %	RT, min	IP, %	Content, %	RT, min	IP, %	Content, %	RT, min	IP, %
**Fatty acids and their esters [FAE]**									
Hexadecanoic acid, ethyl ester (palmitic acid ester)	5.79	37.206	90	6.52	37.203	89	7.22	37.223	85
Octadecanoic acid, ethyl ester	–	–	–	–	–	–	2.34	40.8	67
9,12-Octadecadienoic acid, ethyl ester (linoleic acid ester)	4.20	41.087	87	8.30	41.089	85	8.39	41.099	82
9,12,15-Octadecatrienoic acid, ethyl ester, (*Z,Z,Z*)-	6.27	41.586	92	–	–	–	8.96	41.599	86
Ethyl 9,12,15-octadecatrienoate	–	–	–	9.94	41.59	90	–	–	–
**SUM**	**16.26**	**–**	**–**	**24.76**	**–**	**–**	**26.91**	**–**	**–**
**Phosphoric acid esters [PAE]**									
Phosphoric acid, diethyl octyl ester	–	–	–	0.92	29.856	70	–	–	–
**SUM**	–	–	–	**0.92**	–	–	–	–	–
**Carbohydrates and their derivatives [CHD]**									
Sucrose	8.41	31.463	74	10.52	31.393	72	–	–	–
**SUM**	**8.41**	**–**	**–**	**10.52**	**–**	**–**	**–**	**–**	**–**
**Hydroxyamino acids derivatives [HAAD]**									
(±)-threo-4-Hydroxy-l-lysine lactone	–	–	–	4.60	26.284	68	–	–	–
**SUM**	–	–	–	**4.60**	–	–	–	–	–
**Carboxylic acids and their derivatives [CAD]**									
Formamide, N-methoxy-	–	–	–	–	–	–	16.96	7.879	69
Propanoic acid, 2-oxo-, methyl ester	2.35	9.735	85	–	–	–	–	–	–
**SUM**	**2.35**	–	–	–	–	–	**16.96**	–	–
**Nitrogen-containing compounds [NCC]**									

1-Pyrrolidinylacetonitrile	–	–	–	1.94	14.992	77	–	–	–
4-Piperidinamine, N,1-dimethyl-	–	–	–	1.36	24.94	67	–	–	–
Benzeneethanamine, 2-fluoro-β,3-dihydroxy-N-methyl-	–	–	–	–	–	–	0.48	32.134	69
**SUM**	–	–	–	**3.30**	–	–	**0.48**	–	–
**Alcohols, aldehydes, esters and their derivatives [AAED]**									
Glycerin	10.07	16.861	87	–	–	–	–	–	–
3-(α-Hydroxyethyl)-aniline	1.07	20.325	70	–	–	–	–	–	–
**SUM**	**11.14**	–	–	–	–	–	–	–	–
**Phenolic compounds [PC]**									
Phenol, 3,5-bis(1-pyrrolidinyl)-	1.16	38.427	70	2.11	38.428	66	–	–	–
Phenol, 4,4'-(1-methylethylidene)bis-	5.77	47.089	68	–	–	–	12.96	47.108	67
Phenol, 2,2'-methylenebis[6-(1,1-dimethylethyl)-4-methyl-	5.78	48.566	88	7.45	48.569	87	–	–	–
**SUM**	**12.71**	–	–	**9.56**	–	–	**12.96**	–	–
**Flavonoids**									
2-(3,4-Dimethoxyphenyl)-6-methyl-3,4-chromanediol	1.93	32.95	77	–	–	–	3.45	32.992	70
**SUM**	**1.93**	–	–	–	–	–	**3.45**	–	–
**Alkane hydrocarbons [AHC]**									
Tetracosane	1.92	42.44	89	–	–	–	–	–	–
**SUM**	**1.92**	–	–	–	–	–	–	–	–
**Organoheterocyclic compounds [OHCC]**									
1,2,4,5-Tetroxane, 3,3,6,6-tetramethyl-	–	–	–	–	–	–	5.17	9.655	68
5,10-Diethoxy-2,3,7,8-tetrahydro-1H,6H-dipyrrolo[1,2-a:1,2-d]pyrazine	–	–	–	1.02	44.158	67	–	–	–
Dodecahydro-7a,11b-diaza-benzo[de]anthracene	2.03	38.962	77	5.36	38.966	83	5.53	38.977	77

Pyridine	17.45	7.148	92	–	–	–	–	–	–
**SUM**	**19.48**	–	–	**6.38**	–	–	**10.70**	–	–
**Dicarboxylic derivatives of benzene [DCDB]**									
1,3-Benzenedicarboxylic acid, bis(2-ethylhexyl) ester	0.45	50.735	70	–	–	–	–	–	–
**SUM**	**0.45**	–	–	–	–	–	–	–	–
**Ubiquinones**									
γ-Tocopherol	1.16	56.895	78	0.79	56.899	70	0.71	56.901	64
**SUM**	**1.16**	–	–	**0.79**	–	–	**0.71**	–	–
**Sterols**									
β-Sitosterol	–	–	–	7.14	53.688	66	–	–	–
**SUM**	–	–	–	**7.14**	–	–	–	–	–
**Phthalic acid esters [PhAE]**									
Dibutyl phthalate	1.39	40.289	91	–	–	–	–	–	–
Di-n-octyl phthalate	0.48	48.952	76	–	–	–	–	–	–
Phthalic acid, butyl hept-4-yl ester	–	–	–	1.28	40.289	82	–	–	–
**SUM**	**1.87**	–	–	**1.28**	–	–	–	–	–
**Terpenes**									
Phytol	21.15	39.468	92	27.34	39.467	90	27.84	39.474	86
3,7,11,15-Tetramethyl-2-hexadecen-1-ol	–	–	–	3.41	32.666	82	–	–	–
Squalene	1.16	51.006	69	–	–	–	–	–	–
**SUM**	**22.32**	–	–	**30.75**	–	–	**27.84**	–	–

Notes: RT – retention time; IP – identification probability.

Table S8. Adaptive changes in metabolomic profile of soil-grown *N. schoberi*.

Compound	Control			30 °C			3 °C		
	Content, %	RT, min	IP, %	Content, %	RT, min	IP, %	Content, %	RT, min	IP, %
**Fatty acids and their esters [FAE]**									
Hexadecanoic acid, ethyl ester (palmitic acid ester)	6.57	37.211	88	6.52	37.203	89	7.63	37.204	91
Octadecanoic acid, ethyl ester	1.59	40.791	70	–	–	–	1.13	40.788	77
(E)-9-Octadecenoic acid ethyl ester	–	–	–	1.57	40.785	75	–	–	–
9,12-Octadecadienoic acid, ethyl ester (linoleic acid ester)	5.58	41.09	87	5.37	41.086	89	7.08	41.087	89
Ethyl 9,12,15-octadecatrienoate	11.28	41.588	91	8.63	41.588	90	–	–	–
9,12,15-Octadecatrienoic acid, ethyl ester, (*Z,Z,Z*)-	–	–	–	–	–	–	13.38	41.586	93
9,12,15-Octadecatrienoic acid, methyl ester, (Z,Z,Z)-	–	–	–	–	–	–	1.13	48.089	69
**SUM**	**25.02**	–	–	**22.09**	–	–	**30.35**	–	–
**Carbohydrates and their derivatives [CHD]**									
Sucrose	–	–	–	12.69	31.48	73	12.19	31.505	75
D-Allose	–	–	–	2.29	32.814	65	–	–	–
**SUM**	–	–	–	**14.98**	–	–	**12.19**	–	–
**Carboxylic acids and their derivatives [CAD]**									
Formamide, N-methoxy-	9.93	7.917	67	–	–	–	–	–	–
Propanoic acid, 2-oxo-, methyl ester	2.23	9.676	73	0.87	9.747	73	2.08	9.767	74
Oxalic acid, dodecyl neopentyl ester	9.51	47.098	70	–	–	–	–	–	–
**SUM**	**21.67**	–	–	**0.87**	–	–	**2.08**	–	–
**Nitrogen-containing compounds [NCC]**									
Propane, 2-nitro-	–	–	–	1.41	17.396	67	–	–	–
**SUM**	–	–	–	**1.41**	–	–	–	–	–

**Alcohols, aldehydes, esters and their derivatives [AAED]**									
2-Propanol, 1-methoxy-	–	–	–	2.04	8.02	64	–	–	–
Diethoxymethyl acetate	–	–	–	–	–	–	2.09	8.079	65
**SUM**	–	–	–	**2.04**	–	–	**2.09**	–	–
**Phenolic compounds [PC]**									
Phenol, 2,2'-methylenebis[6-(1,1-dimethylethyl)-4-methyl-	27.46	48.57	91	1.58	48.566	81	–	–	–
**SUM**	**27.46**	–	–	**1.58**	–	–	–	–	–
**Flavonoids**									
2-(3,4-Dimethoxyphenyl)-6-methyl-3,4-chromanediol	2.65	31.395	72	4.59	32.95	76	2.90	32.95	76
**SUM**	**2.65**	–	–	**4.59**	–	–	**2.90**	–	–
**Alkane hydrocarbons [AHC]**									
Tetratetracontane	–	–	–	0.99	52.588	70	–	–	–
**SUM**	–	–	–	**0.99**	–	–	–	–	–
**Organoheterocyclic compounds [OHCC]**									
Pyridine	–	–	–	9.64	7.184	93	7.36	7.197	94
8-Azabicyclo[3.2.1]oct-6-en-3-one, 8-methyl-	–	–	–	0.66	20.328	70	–	–	–
6-Methyl-1,2,3,4-tetrahydroquinoline	–	–	–	0.72	29.197	68	–	–	–
5,10-Diethoxy-2,3,7,8-tetrahydro-1H,6H-dipyrrolo[1,2-a:1',2'-d]pyrazine	–	–	–	0.67	44.153	68	0.64	44.159	74
Cyclohexane, isocyanato-	1.14	13.993	81	–	–	–	–	–	–
Dodecahydro-7a,11b-diaza-benzo[de]anthracene	2.87	38.969	80	4.59	38.959	84	3.51	38.961	85
Oxazole, 5-hexyl-2,4-dimethyl	–	–	–	–	–	–	1.56	39.771	69
**SUM**	**4.01**	–	–	**16.28**	–	–	**13.07**	–	–
**Ubiquinones**									
γ-Tocopherol	–	–	–	1.65	56.894	68	1.84	56.893	82

**SUM**	–	–	–	**1.65**	–	–	**1.84**	–	–
**Phthalic acid esters [PhAE]**									
Dibutyl phthalate	2.96	40.29	88	–	–	–	–	–	–
**SUM**	**2,96**	–	–	–	–	–	–	–	–
**Terpenes**									
Phytol	14.63	39.468	90	31.88	39.465	93	34.13	39.467	93
Squalene	1.60	51.012	66	1.64	51.007	68	1.35	51.01	70
**SUM**	**16.23**	–	–	**33.52**	–	–	**35.48**	–	–

Notes: RT – retention time; IP – identification probability.
